# Deciphering the relational dynamics of AF-2 domain of PAN PPAR through drug repurposing and comparative simulations

**DOI:** 10.1371/journal.pone.0283743

**Published:** 2023-03-31

**Authors:** Fouzia Gul, Nousheen Parvaiz, Syed Sikander Azam

**Affiliations:** Computational Biology Lab, National Center for Bioinformatics (NCB), Quaid-i-Azam University, Islamabad, Pakistan; Gauhati University, INDIA

## Abstract

Peroxisome proliferator-activated receptors (PPARs) are nuclear receptors, and their activation has been proven to treat mild liver fibrosis, reduce steatosis, inflammation, and the extrahepatic effects of chronic liver disease. Considering the significance of the PPARs, it is targeted for the treatment of Non-Alcoholic Steatohepatitis (NASH), for which currently there is no FDA-approved drug. Lanifibranor is a next-generation highly potential indole sulfonamide derivative that is presently in clinical trial phase III as an anti-NASH drug which fully activates PPARα and PPARδ and partially activates PPARγ. In the current study, a comprehensive computational investigation including 3D-QSAR pharmacophore modeling, MD simulations and binding free energy calculations is performed to get insights into the activation mechanism of the Lanifibranor. Furthermore, FDA-approved drugs were explored for repurposing through virtual screening against each PPAR pharmacophore to identify potential drug candidates. Forasartan, Raltitrexed, and Lifitegrast stood out as potential agonists for PPARα (full agonist), PPARγ (partial agonist), and PPARδ (full agonist), respectively. The findings of the study highlighted a lack of hydrogen bond acceptor feature in Raltitrexed and Lanifibranor which is responsible for partial activation of PPARγ that plays a critical role in preventing lipid accumulation. In addition to this, the significant role of AF2 domain in full and partial activation of PPARs through electrostatic interactions was also revealed, that facilitates the anchoring of ligand within the binding cavity. Moreover, common chemical scaffolds (methyl sulfonyl benzene, butyric acid, and chlorobenzene) identified using Fingerprinting technique were presented in this study which hold the potential to aid in the design and development of target specific novel Pan PPAR medications in future.

## 1. Introduction

Non-Alcoholic Steatohepatitis (NASH) is a potentially fatal chronic liver disease. NASH is an emerging public health issue, characterized by inflammation, hepatocellular lipid buildup, liver cell injury in the form of hepatocyte ballooning, and steatosis both with or without fibrosis in lack of excessive alcohol intake [1–3]. It is a widespread multifactorial and multi-stage liver disease that can proceed to hepatocellular carcinoma or cirrhosis and end-stage liver disease i.e. liver failure resulting in high morbidity and mortality rates [4–8]. It has also been identified as a major cause in patients evaluated for liver transplantation, with higher cardiovascular risk and malignancy being observed in these patients [9–11]. The precise source of its pathogenicity is unclear due to the factors linked with the fast progression that could not be distinguished [12, 13]. The major hypothesis is centered around the role of certain conditions such as genetic predisposition, thyroid-stimulating hormone levels abnormal lipid metabolism, oxidative stress, lipo-toxicity, mitochondrial dysfunction, altered production of cytokines and adipokines, gut dysbiosis, endoplasmic reticulum stress, and glucotoxicity that are predictors of histologic findings diagnostic of NASH [14–20].

Despite continuous progress in understanding the pathogenesis of NASH, finding potential therapeutic targets, and progressing drug development, there are substantial unresolved challenges, and there is presently no FDA-approved drug for NASH [21, 22], therefore the urgent need for an effective therapy that addresses the complicated pathophysiologic mechanisms of NASH can no longer be ignored [23]. Numerous research initiatives that were specifically designed to treat NASH have shown promising results initially but were halted in the late phase of trials owing to ineffectiveness, safety issues, or drug-drug interactions [24]. Given the numerous targets that may be associated in NASH, many compounds now under research are considerable. A potential drug for NASH would be the one that targets fat deposition, emphasizes anti-metabolic activities, has anti-fibrotic and anti-inflammatory characteristics, and minimizes cardiovascular risk, which is the primary cause of death in NASH [25, 26].

Peroxisome Proliferator activated receptor (PPARs), a therapeutic target that functions as a master regulator in the liver and adipose tissue has gained prominence over recent years. The deregulation of PPAR accelerates the progression of NASH by influencing inflammation, lipid metabolism, insulin resistance, and fibrogenesis. PPARs are activated by ligands and bind to fatty acids. It belongs to the Nuclear Hormone receptors superfamily which play an essential role in whole-body energy metabolism [27, 28]. The ligand activated PPAR forms a heterodimer by binding to the retinoid X receptor (RXR). This heterodimer further binds to PPREs (PPAR response elements) of targeted genes in the promoter region resulting in the transactivation of mitochondrial and peroxisomes target genes [29, 30]. PPAR has three different isoforms, namely: PPAR-Alpha (NR1C1), PPAR-Gama (NR1C3), and PPAR-Delta (NR1C2). PPARs are different from each other in the spectrum of their distribution, functionality, and ligand specificity, however, they target the same segment of DNA. The sequence comparison of all isotypes shows high similarity in the DNA binding domains (DBD) which means that DNA binding domains are extremely conserved whereas the Ligand-binding domains (LBD) are less conserved [31]. Some conserved LBD residues have been linked to essential receptor activity engaged in signal transduction. The substantial variation in the LBD residues shows that each receptor isotype is pharmacologically different.

PPARα expresses in the adipose tissue, liver, kidney, heart, and skeletal muscle [32]. It also has an anti-inflammation effect. The activation of PPARα increases the activity of the lipoprotein lipase (LPL) by upregulation of gene transcription and by reducing the level of apolipoprotein (apo) C-III, which is a natural inhibitor of LPL. Through these combined actions the triglyceride-rich lipoproteins TRL levels decreases [33]. PPARα activation also reduces weight gain by improving lipid and glucose metabolism. PPARγ is primarily expressed in adipose tissue where it regulates energy balance, adipogenesis, and lipid biosynthesis [34]. It is also expressed in the colon, the immune system, and the retina to some extent. This receptor participates in the accumulation of lipids in adipose tissue and insulin sensitivity [34, 35]. The PPARδ express in adipose tissue, skeletal muscle, skin, and muscles where it regulates the fatty acid beta-oxidation and mitochondrial metabolism [36, 37]. PPARδ activation improves glucose tolerance by increasing fatty acid oxidation and energy expenditure, indicating a role in inflammation and fibrosis [36].

**Fig 1:**
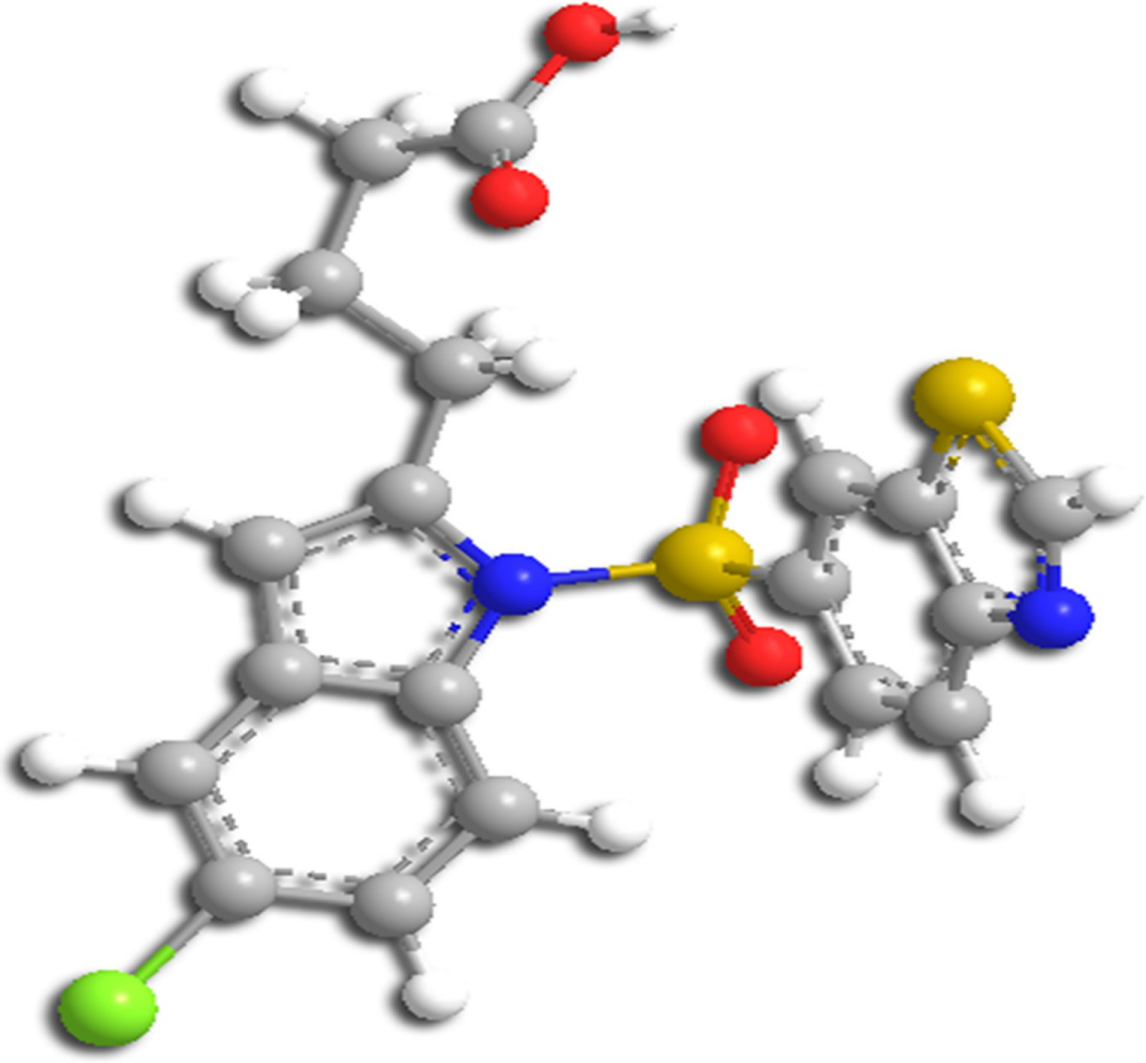
Three-dimensional structure of Lanifibranor.

Previous studies have demonstrated that activating one or more PPAR isoforms has therapeutic benefits in preclinical models of liver damage. PPAR activation has been shown to cure moderate liver fibrosis, decrease steatosis, inflammation and alleviate the extrahepatic consequences of chronic liver disease [38, 39]. However, in the clinical relevance to NASH, none of these studies have looked at the effects of activating all three PPAR isoforms simultaneously [40, 41]. The current study, in this regard, has therefore focused on the efficacy of Lanifibranor, a drug designed by Inventiva and synthesized by Boutia et al. (Fig 1). It is a next-generation highly potential indole sulfonamide derivative, an anti-NASH drug, currently in the clinical trial phase III [42] designed to target and well-balanced activation of all three subtypes of PPAR shown to act on PPARα, PPARγ, and PPARδ with an EC50 value of 1.5, 0.21, and 0.87μM respectively [43]. Given the critical role of the PPARs, it is not unexpected that this nuclear receptor family has been the subject of therapeutic research for the treatment of metabolic diseases such as NASH. In this study, we have generated 3D-QSAR pharmacophore, performed virtual screening, molecular docking studies and molecular dynamic simulations of top docked compounds from the FDA-approved library against each PPAR and Lanifibranor, to find out detailed structural dynamic information and activating mechanism. The binding of Lanifibranor to each PPAR was identified and described, supporting Lanifibranor’s action as a well-balanced Pan PPAR agonist. Furthermore, structural dynamics of protein-ligand complexes have been explored to elucidate the underlying mechanism for completely activating PPARα and PPARδ as well as partially activating PPARγ.

## 2. Materials and methods

**Fig 2:**
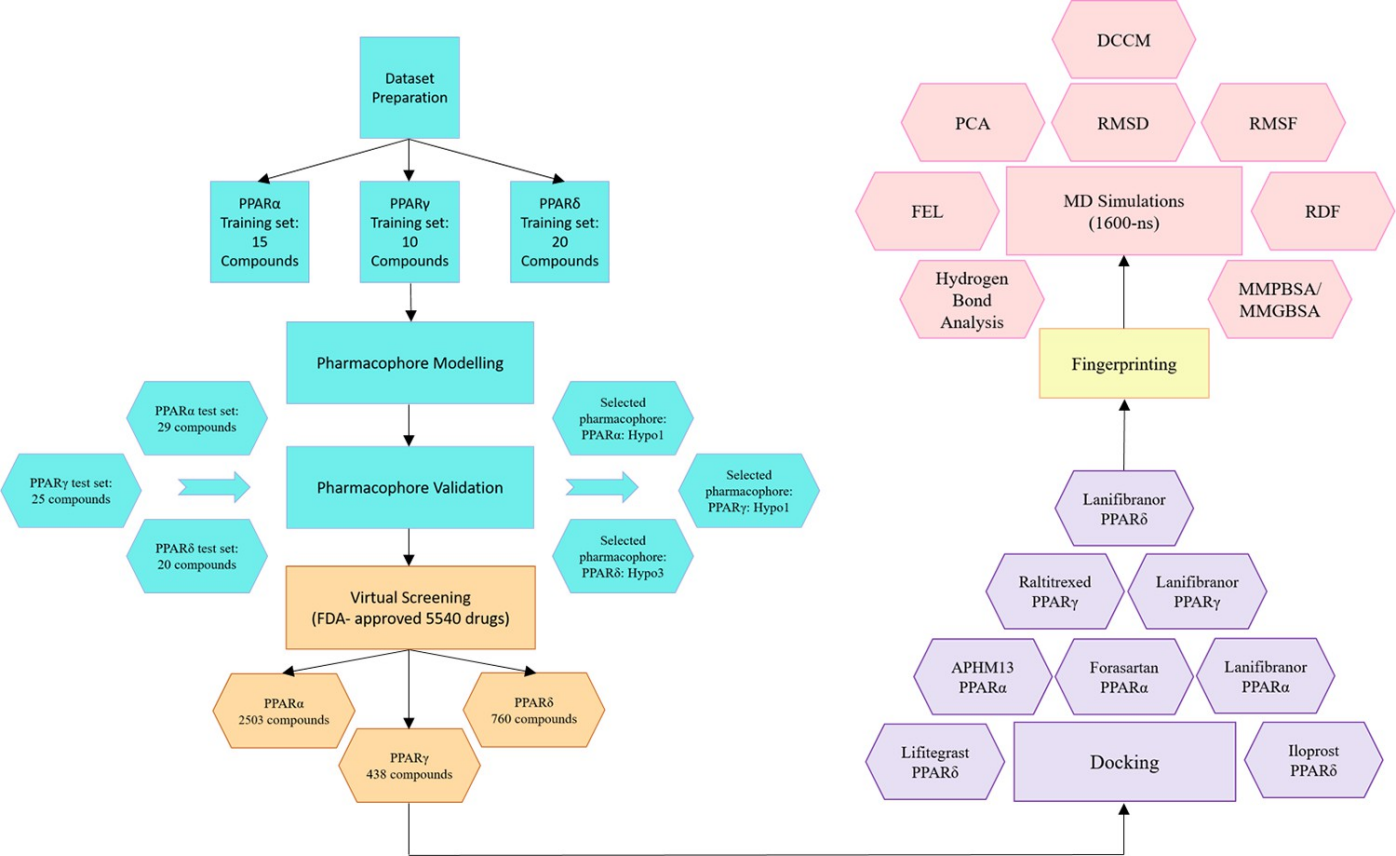
The overall methodology followed in current research work.

The overall computational approaches employed in this comprehensive research study are illustrated below in Fig 2.

### 2.1. Dataset preparation

Datasets were collected for each PPAR from the identified and reported agonists. A dataset of 43 compounds for PPARα, 39 compounds for PPARγ, and 45 compounds for PPARδ were prepared. The compound’s agonist activity was expressed as EC50 (i-e. 50% of the maximum effect of a compound is exhibited at this concentration). The datasets were categorized into two sets (active and inactive) based on EC50 values. The compounds having EC50 values ranging from 0.0001 μM to 0.35 μM were kept in the active category and all the remaining were in the inactive category. The top 15 active compounds of PPARα (S1 Table), top 10 compounds of PPARγ (S2 Table), and top 20 active compounds of PPARδ (S3 Table) from the collected dataset were taken as a training set to build a model and all the remaining compounds were taken as a test set for pharmacophore mapping and hypothesis validation. The training set includes the most active compounds, and the test set includes both active and inactive compounds.

### 2.2. Pharmacophore modelling

The pharmacophore model was generated using the three-dimensional quantitative structure-activity relationship (3D-QSAR) based pharmacophore approach. The HypoGen module of Discovery Studio® (DS) [44], was used to generate a hypothesis that utilizes the chemical features found in active compounds but not in inactive compounds. The feature mapping protocol provided in DS was used to identify the common features of training sets. As determined by feature mapping protocol, ring aromatic (RA), hydrogen bond acceptor (HBA), hydrophobe (HY), and negative ionizable (NI) features were mapped on the training set of all three subtypes of PPAR.

Above mentioned features were used to generate 10 hypotheses with the minimum of zero to the maximum of 5 features for each PPAR utilizing the 3D-QSAR protocol of DS. For hypothesis generation, the energy threshold for conformational generation for each compound was maintained at 10 kcal mol^-1^. The Minimum Interfeature Distance and maximum excluded volume were set to 1.5 and zero, respectively. As defined by DS, the uncertainty value that is the ratio of minimum and maximum value of the reported value was set at 1.5.

### 2.3. Validation of pharmacophore model

The pharmacophore model was validated using the test sets to investigate the capacity of the generated models. For validating pharmacophore models. The test consisted of both active and inactive compounds for PPARα (28 compounds), PPARγ (29 compounds), and PPARδ (25 compounds). The best pharmacophore model was selected from the 10 generated hypotheses based on high fit value, cost analysis, high correlation, and lowest root mean square deviation (RMSD) using the Catalyst/HypoGen module of DS. The fit value shows the quality of the mapping of compounds to the hypothesis. The cost difference is the difference between the total cost and the null cost. The overall cost of a good hypothesis is near to fixed cost and far from the null cost. The similarity and closeness in the data set with each other are measured by the correlation coefficient. The highest the correlation coefficient, the highest the similarity, and more closeness in the data set. The lower the RMSD value, the better superimposition of structures over pharmacophore models. By applying the above-mentioned statistical parameters, the best model of pharmacophore was selected. To evaluate the pharmacophore model’s prediction power, all compounds in the test set were mapped to the hypothesis model using the Ligand Pharmacophore Mapping protocol in DS.

### 2.4. Virtual screening

For virtual screening library of FDA-approved drugs containing 5540 compounds was used. Using the screen library protocol of DS, this library of compounds was screened against the best-validated pharmacophore model of each PPAR. The optimizing features parameters were set as 3 for minimum and 4 for maximum. The three feature best hits and all four features’ hits resulted from the virtual screening against their respective pharmacophore was docked to their relative subtype receptor.

### 2.5. Molecular docking

For docking, the crystal structures of subtypes of Peroxisome Proliferator-Activated Receptor: PPARα (PDB ID: 3VI8), PPARγ (PDB ID: 6ENQ) and PPARδ (PDB ID: 3SP9) were acquired from Research Collaboratory for Structural Bioinformatics Protein Data Bank (RCSB PDB) in pdb format. The proteins were prepared for docking and dynamic studies. The PPARα and PPARγ were modeled due to the missing residues using PDB ID: 3VI8 and 6ENQ as a template, respectively. The residues at Ω-loop 196–202(3VI8) and 260–275 (6ENQ) were missing. The Ω-loop is a highly flexible and disordered region of LBD of PPARs, due to which it remained unmodelled. The Ω-loop works as a gate to the ligand binding pocket and moves substantially during the conformational rearrangement that accompanies ligand binding to the LBD [45]. The modeled structures were energy minimized using University of California San Francisco (UCSF) Chimera [46] for 1500 total steps which were divided into the first 750 steps of steepest descent and last 750 steps of the conjugate gradient. The step length in all steps was kept at 0.02 (default). During minimization, for standard residues, the Amber (Assisted model building with energy refinement) parameters were used, and the Antechamber module was used to assign parameters for non-standard residues.

The LibDock protocol under the protein-ligand interaction section in DS was used for molecular docking. The LibDock is a high-throughput algorithm [47]. The Lanifibranor, the FDA-approved compounds (4 feature all hits and 3 feature best hits) obtained from virtual screening, and all three-crystal ligands were docked into its respective receptor (3VI8 (PPARα), 6ENQ (PPARγ), and 3SP6 (PPARδ)). The 3 feature best hits and 4-feature all hits were docked PPARα (2510 drugs), PPARγ (444 drugs), and PPARδ (765 drugs), obtained from virtual screening. The docking protocol was set to default. All docked poses were rated and categorized based on the LibDock score, and all compounds were ranked based on the LibDock score.

### 2.6. Similarity search

In the field of cheminformatics to screening similar molecules is a smart practice, as the assumption underlies a fundamental principle that chemical compounds with similar structures should elicit similar biological activities [48]. The ‘Find Similar Molecules by Fingerprints’ protocol in DS provides a Tanimoto coefficient (Tc), which was adopted as the evaluation criterion to find similarity of ligands in an input library with the reference ligand that is Lanifibranor as it is a single drug that targets PPAR altogether. The cut-off value of 50% was taken for similarity search.


Tanimoto:SA/(SA+SB+SC) (1)

In the above Tanimoto equation, the SA represents the number of bits that are present in both the target and the reference, the SB is the number of bits that are present in the target but not in the reference, and the SC denotes the number of bits in the reference but not the target.

### 2.7. Molecular dynamics simulations

Molecular dynamic simulation is performed to explore the conformational space of proteins, particularly intermediate states or transitory states that play significant roles in the ligand-protein binding and unbinding. Molecular docking can also be used to determine the binding mechanism of a protein and its ligand. However, the MD simulations not only improve the local steric clashes between protein and ligand, yet also correct and optimize the ligand’s initial mode during molecular docking. Sixteen hundred nanoseconds (ns) simulation was done to study the dynamic behavior of the complex utilizing the AMBER force field [49].

Systems were prepared using an antechamber program of AMBER. The systems were solvated by placing the complexes in a cubic box of 12 Å with a three-point convertible intermolecular potential (TIP3P) water box. The minimization was performed by imposing a 200 kal/mol constraint on the hydrogen atoms for 500 steps, followed by 1000 steps of minimization for the water box. Using Langevin dynamics, the entire system was heated to 300 K at 1 atm for 20 picoseconds and maintained at that temperature [50]. The SHAKE algorithm was applied to constraints the bonds involved between hydrogen atoms and heavy atoms, and the NVT ensemble was used for heating [51]. The system pressure was maintained with a time scale of 50-ps by the NPT ensemble. When calculating non-bonded interactions using the Berendsen method with NVT ensemble for a production run of 200 ns per system, a cut-off radius of 8.0 was applied. The AMBER trajectory analysis tool CPPTRAJ was used to evaluate system simulation trajectories [52].

### 2.8. Hydrogen bond analysis

The hydrogen bond plot in Amber was produced depending on time using the cpptraj module. As a default value, a fraction of donor and acceptor atoms of ≥ 0.05 Å was set. The colored lines in the plot show the residues engaged in strong hydrogen bonding throughout the simulation time of 200 ns.

### 2.9. Radial distribution function

A ligand’s or molecule’s structural assessment and distribution with a reference protein atom or residue was determined by the Radial Distribution Function (RDF) [53]. RDF is the probability of finding a group of N atoms of ligand in a given spherical volume of radius r at a specific distance from another specific atom of the protein [54]. The PTRAJ module of AMBER was used to display and study the conformational changes caused by molecular interactions between the active site residues and ligands. The radial distribution function is represented as:

$$g(r)=\frac{\rho ij(r)}{<\rho j>}=\frac{nij(r)}{<\rho j>4\pi r\delta r}$$ (2)

In this equation, g(r) is defined as a ratio of the observed number density *ρij* to average number density *ρj* at the distance r. While *nij* donates the number of atoms in specified volume and factor *4πrδr* measures the shell volume of a spherical with thickness *δr*.

### 2.10. Binding free energy calculation

MMPBSA and MMGBSA methods were employed for binding free energy calculation (BFEC) [55]. Both these methods are very closely related. These methods sum up solvation free energy G_solv_, gas phase energy G_gas_, electrostatic interactions, and van der Waal energies [56]. The prediction of binding free energy of ligand to the receptor is of great importance in computational biology as it can be used to identify the novel molecule that can bind to a target and act as a therapeutic drug [56]. The binding energy of all complexes was calculated through MMGBSA/MMPBSA method using the module, MMPBSA.py in AMBER. Topology files (prmtop) of ligand, receptor, and complex were created using the Ante-MMPBSA.py module. Binding free energy decomposition per residue was computed using Van-der Waals energy, electrostatics interactions, polar solvation energy, and non-polar solvation energy for residues with the binding energies equivalent to and greater than 1 kcal mol^-1^ [57]. The difference between the complex free energies of the receptor and the ligand is calculated using the total binding energy equation.


$$\Delta G_{bind}=G_{complex}-[G_{receptor}+G_{ligand}]$$ (3)

### 2.11. Principal component and free energy landscape analysis

Principal component analysis (PCA) was used to achieve understanding of the internal motion of the system to comprehend the motion of MD trajectories [58, 59] using CPPTRJ module of Amber. Employing orthogonal coordinate transformation, a diagonal matrix of eigenvalues was produced to obtain the spatial covariance matrix for the eigenvectors and their atomic coordinates. The principal components were generated using the eigenvectors and eigenvalues. The dominating movements throughout the simulation were plotted utilizing these PCs [60, 61]. The following equation was used to determine the free energy landscape (FEL) using the first two principal components (PC1 and PC2).

$$\Delta G(X)={-K}_{B}TlnP(X)$$ (4)

where X indicates the response of the two principal components, KB is the Boltzmann constant, and P(X) is the dispersion of the framework’s likelihood on the first two principal components.

### 2.12. Dynamic cross-correlation matrix

A 3D matrix depiction of amino acid residue motion over time. This method analyses Cα atoms across the correlation matrix for all complexes to determine continuous correlations. The Dynamic Cross-Correlation Matrix (DCCM) was investigated via ProDy [62] and To illustrate the results, Matplotlib was utilized [63]. DCCM values range between -1 and +1 in which a value greater than zero signifies the positive correlation motion among two atoms and a value that is less than zero signifies the negative correlation motion. The DCCM plot illustrates both positive (same direction) and negative correlation (opposite direction). When the receptor and ligand interact, a positive correlation depicts that their motions are parallel, and the system shows stability. Contrarily, a negative correlation suggests the instability in the complex or that the ligand is moving out of the binding pocket, causing an anti-parallel correlation. In addition to this, the strength of the positive and negative correlations is proportional to the intensity of the colors in the DCCM map. The positive correlation is indicated by red, whereas negative correlation is depicted by blue; a darker color implies a more meaningful association, and vice versa.

## 3. Results and discussions

### 3.1. Pharmacophore modeling

The pharmacophore model for PPARα, PPARγ, and PPARδ was generated using the diverse range of training sets.

#### 3.1.1. Pharmacophore for PPARα

**Fig 3:**
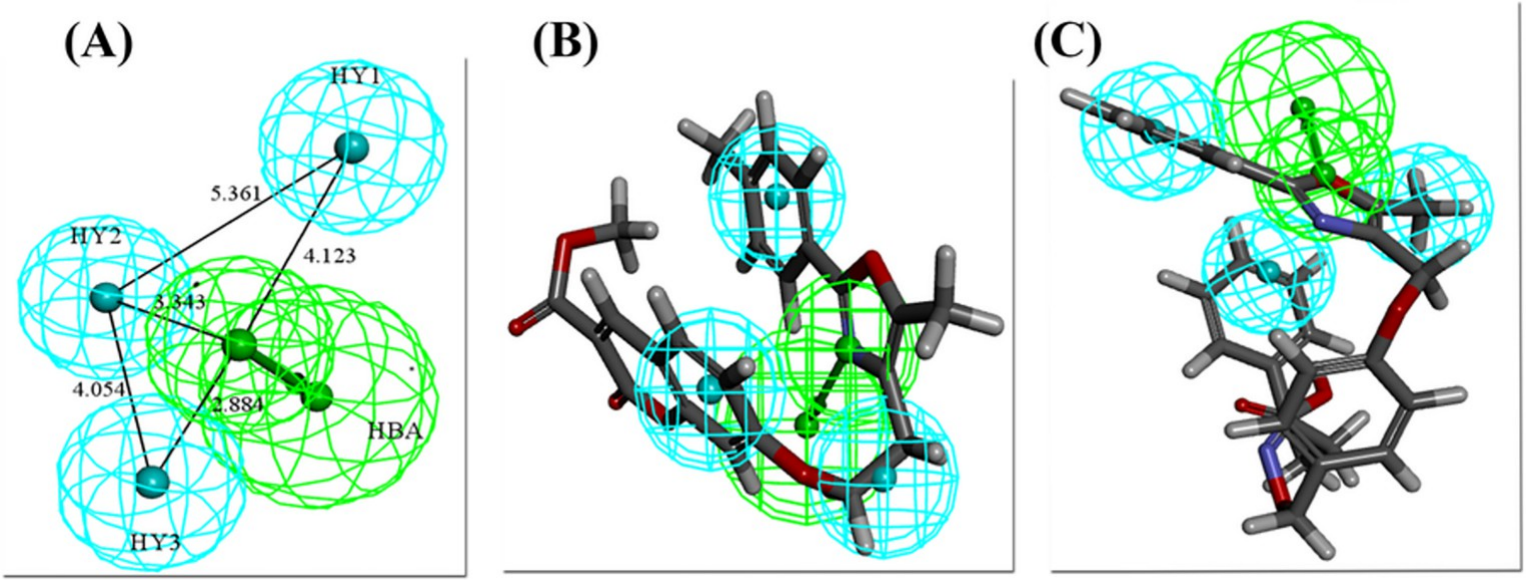
(A) Best pharmacophore for PPARα (hypo1) with distance labeled chemical features. (B) The most active compound of the training set mapped on the pharmacophore model. (C) The least active compound of the training set mapped on the pharmacophore model.

The training set comprised of 15 compounds generated 10 hypotheses for PPARα. The details of the generated hypotheses for PPARα are listed in Table 1. The hypo1 was chosen as the best hypothesis based on the statistical criteria. The hypo1 has the highest cost difference of 56.678, the highest maximum fit of 7.07, the lowest root means square deviation (RMSD) of 1.1 and the highest correlation coefficient of 0.945. The fixed cost, null cost, and total cost for the hypo1 were 55.4959, 121.658, and 64.9796 respectively. The hypo1 of PPARα contains 4 features: 1 HBA and 3 HY. These features were mapped on all PPARα training set compounds using the ligand pharmacophore mapping protocol of DS. The most active and the least active of the training set of PPARα mapped on the selected pharmacophore with all features has exhibited the fit score of 7 and 6.3, respectively (Fig 3).

**Table 1:** Statistical parameters of top 10 pharmacophore hypotheses generated with HypoGen algorithm for PPARα.

Hypothesis	Features	Maximum fit	Total cost	Cost difference	RMSD	Correlation
**Hypo1**	**HBA, 3HY**	**7.07**	**64.97**	**56.678**	**1.10**	**0.945**
Hypo2	HBA, 3HY	7.45	66.44	55.210	1.20	0.934
Hypo3	HBA, HY, 2RA	7.21	68.75	55.901	1.31	0.921
Hypo4	2HBA, 2HY	8.34	69.14	52.513	1.34	0.917
Hypo5	2HBA, HY, RA	7.59	69.55	52.101	1.36	0.915
Hypo6	2HBA, HY, RA	6.82	69.72	51.933	1.35	0.916
Hypo7	2HBA, HY, RA	6.30	70.71	50.943	1.37	0.913
Hypo8	2HBA, HY, RA	7.07	70.79	50.862	1.41	0.908
Hypo9	2HBA, HY, RA	5.84	70.94	50.708	1.35	0.916
Hypo10	2HBA. RA	4.98	71.18	50.471	1.41	0.908

#### 3.1.2. Pharmacophore for PPARγ

**Fig 4:**
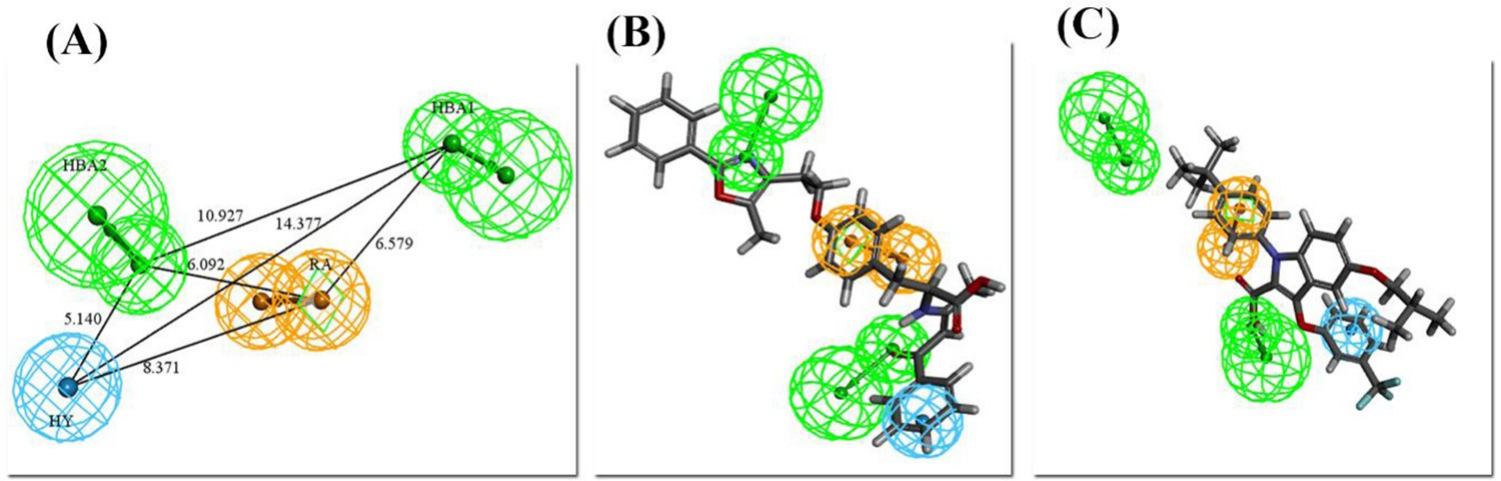
(A) Best pharmacophore for PPARγ (hypo1) with distance labeled chemical features. (B) The most active compound of the training set of PPARγ mapped on the pharmacophore model. (C) The least active compound of the training set of PPPARγ mapped on pharmacophore.

From the 10 hypotheses generated for PPARγ using the training set consisting of 10 compounds, the hypo1 was the best based on statistical parameters. The detail of the generated hypotheses is summarized in Table 2. The hypo1 has the highest cost difference of 53.028, the total cost of 48.277, the highest maximum fit of 8.55, the lowest root mean square deviation (RMSD) of 0.7, and the highest correlation coefficient of 0.98 with the Fixed cost and Null cost of 45.70 and 48.27 respectively. The hypo1 of PPARγ contains four features: 2 HBA, 1 HY-AR, and 1 RA. These features were mapped on all training set compounds of PPARγ. The training sets’ most active compound of PPARγ was mapped on the selected pharmacophore with all features showed the fit score of 8.2 whereas the least mapped with three features (HBA, HY-RA, and RA) and had the fit score of 6.2 (Fig 4).

**Table 2:** Statistical parameters of top 10 pharmacophore hypotheses generated for PPARγ using HypoGen algorithm.

Hypothesis	Features	Maximum fit	Total cost	Cost difference	RMSD	Correlation
**Hypo1**	**2HBA, HY-AR, AR**	**8.55**	**48.27**	**53.028**	**0.70**	**0.984**
Hypo2	2HBA, HY, RA	7.37	48.39	53.914	0.71	0.983
Hypo3	3HBA, HY	7.54	48.91	52.391	0.79	0.979
Hypo4	HBA, 2HY-RA, RA	7.54	49.51	51.792	0.86	0.975
Hypo5	2HBA, HY, RA	8.11	49.84	51.464	0.90	0.973
Hypo6	2HBA, HY, RA	7.13	49.88	51.422	0.88	0.974
Hypo7	2HBA, HY-RA, HY	8.25	50.14	51.159	0.94	0.971
Hypo8	HBA, RA, RA	6.31	50.55	50.750	0.97	0.968
Hypo9	2HBA, HY-RA, HY	7.64	50.80	50.505	1.00	0.966
Hypo10	2HBA, HY-RA, RA	6.76	50.83	50.474	0.95	0.970

#### 3.1.3. Pharmacophore for PPARδ

The detail of the hypotheses that were generated from the training set of 20 compounds for PPARδ is tabulated in Table 3. The hypo3 is selected as the best hypothesis based on its highest maximum fit score i.e. 5.04 and the maximum number of features. The hypo3 has four features: 1 HBA, 2 HY, and 1 NI. The cost difference, total cost, root mean square deviation (RMSD), and correlation coefficient were 22.351, 88.49, 1.30, and 0.856 respectively. The Fixed cost was 64.47 and the Null cost was 110.84. The features of hypo3 were mapped on all training set compounds of PPARδ.

**Table 3:** Statistical parameters of top 10 pharmacophore hypotheses generated for PPARδ using HypoGen algorithm.

Hypothesis	Features	Maximum fit	Total cost	Cost difference	RMSD	Correlation
Hypo1	2HBA, NI	3.49	82.42	28.42	1.00	0.917
Hypo2	HBA, HY, NI	3.77	84.00	26.84	1.11	0.897
**Hypo3**	**HBA, 2HY, NI**	**5.04**	**88.49**	**22.35**	**1.30**	**0.856**
Hypo4	HBA, HY, NI	3.37	90.75	20.09	1.34	0.847
Hypo5	2HBA, NI	3.32	91.27	19.56	1.35	0.843
Hypo6	HBA, 2HY, NI	4.94	91.77	19.07	1.41	0.828
Hypo7	HBA, HY, NI	3.51	92.43	18.10	1.42	0.827
Hypo8	HBA, HY, NI	3.63	93.23	17.60	1.46	0.816
Hypo9	HBA, NI, RA	3.46	93.62	17.22	1.45	0.817
Hypo10	HBA, NI, RA	4.15	93.75	17.08	1.52	0.797

**Fig 5:**
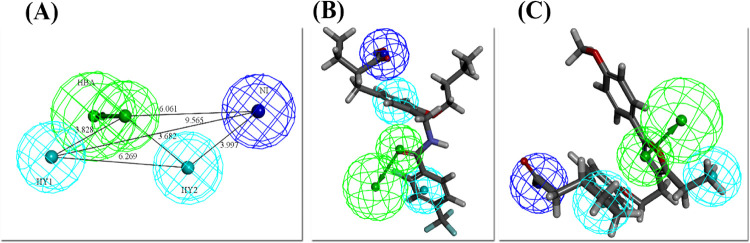
(A) Best pharmacophore for PPARδ (hypo3) with distance labeled chemical features. (B) The most active compound of the training set of PPARδ mapped on the pharmacophore model. (C) The least active compound of the training set of PPARδ mapped on the pharmacophore model.

Feature mapping was applied on the most active and least active compound of the training set for PPARδ selected pharmacophore which mapped with all features showed the fit score of 4.9 and 4.5 respectively (Fig 5).

### 3.2. Mapping of Lanifibranor on pharmacophores

**Fig 6:**
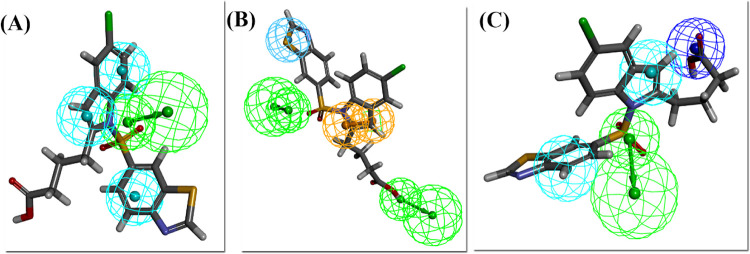
Lanifibranor mapped on pharmacophore for (A) PPARα (B) PPARγ and (C) PPARδ.

Lanifibranor was also mapped on all the three pharmacophores which showed the fit value of 5.0, 6.2, and 4.7 for PPARα, PPARγ, and PPARδ respectively (Fig 6). Lanifibranor mapped on PPARγ pharmacophore with three features (HBA, RA, and HY) leaving one HBA feature unmapped. The generated pharmacophore models highlighted important pharmacophoric characteristics influencing the PPAR activation which were found to be consistent with the reported pattern of biological activity. The features hydrogen bond acceptor (HBA) and hydrophobe (HY) were the common features found amongst all three pharmacophore models for all three subtypes of PPAR respectively. The PPAR has a ligand-binding cavity composed of substantially both polar and hydrophobic regions as reported in the literature, hence making it essential for the ligands to have hydrophobic and hydrogen bond acceptor features [64].

### 3.3. Virtual screening

Virtual screening is considered as an efficient approach in which large libraries of small molecules are screened, that can be used to find potential and novel hits for the development in drug discovery [65]. The compounds having these pharmacophore features were retrieved through virtual screening. The library of FDA-approved drugs containing 5540 compounds was screened against designed pharmacophores. A set of compounds that contained the 3 features best hit and 4- features all hits for PPARα (2503 compounds), PPARγ (438 compounds), and PPARδ (760 compounds) were obtained. The selected hit compounds were then employed for molecular docking analysis against their respective receptor.

### 3.4. Molecular docking analysis

**Fig 7:**
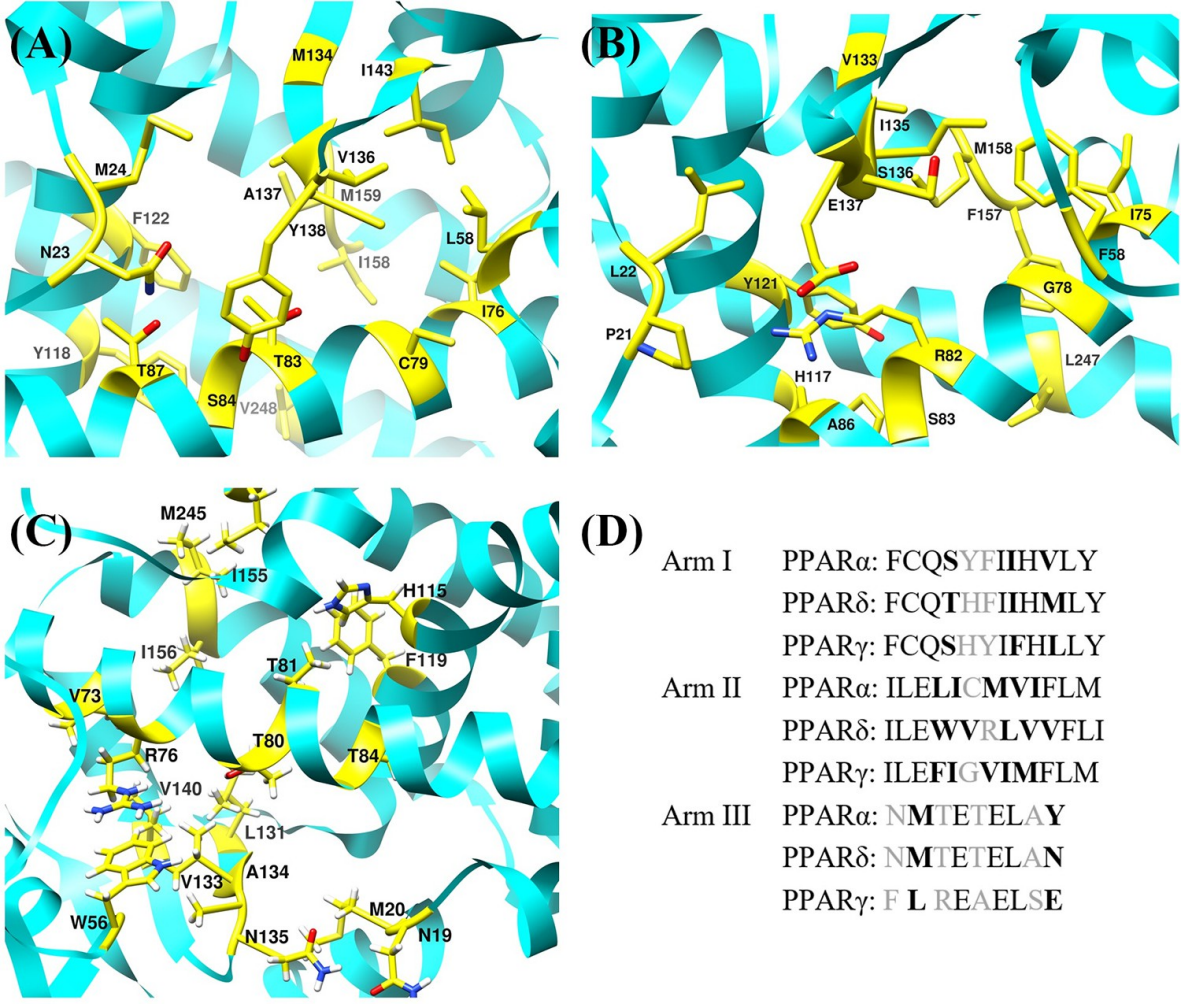
The three-dimensional view of the residues of the binding pocket of (A) PPARα, (B) PPARγ, (C) PPARδ shows the difference between them. (D) The alignment of the binding site residues of the human PPAR subtypes. The black, bold and gray color represents identical residues, residues with same chemical character and residues with different chemical character, respectively [66].

Protein-ligand interactions are crucial in understanding the biological regulatory process and provide a theoretical foundation for the development and identification of novel therapeutic targets. The PPARs have large Y-shaped binding pocket composed of three sub-arms (Arm-I, Arm-II and Arm-III). Arm-I and Arm-II of PPAR show significant homology whereas the Arm-III is less conserved. The difference between the residues of the binding pocket of each PPAR is represented in the Fig 7 along with the alignment of these residues [66]. The crystal ligands (APHM13, Lanifibranor, and Iloprost) and FDA-approved drugs obtained as a result of virtual screening were docked to their respective receptors (PPARα, PPARγ, and PPARδ) and clinical trial drug Lanifibranor was docked to all PPAR subtypes. The FDA-approved drugs that showed the highest LibDock score were selected for molecular dynamics simulation studies.

#### 3.4.1. Crystal ligands docking

**Fig 8:**
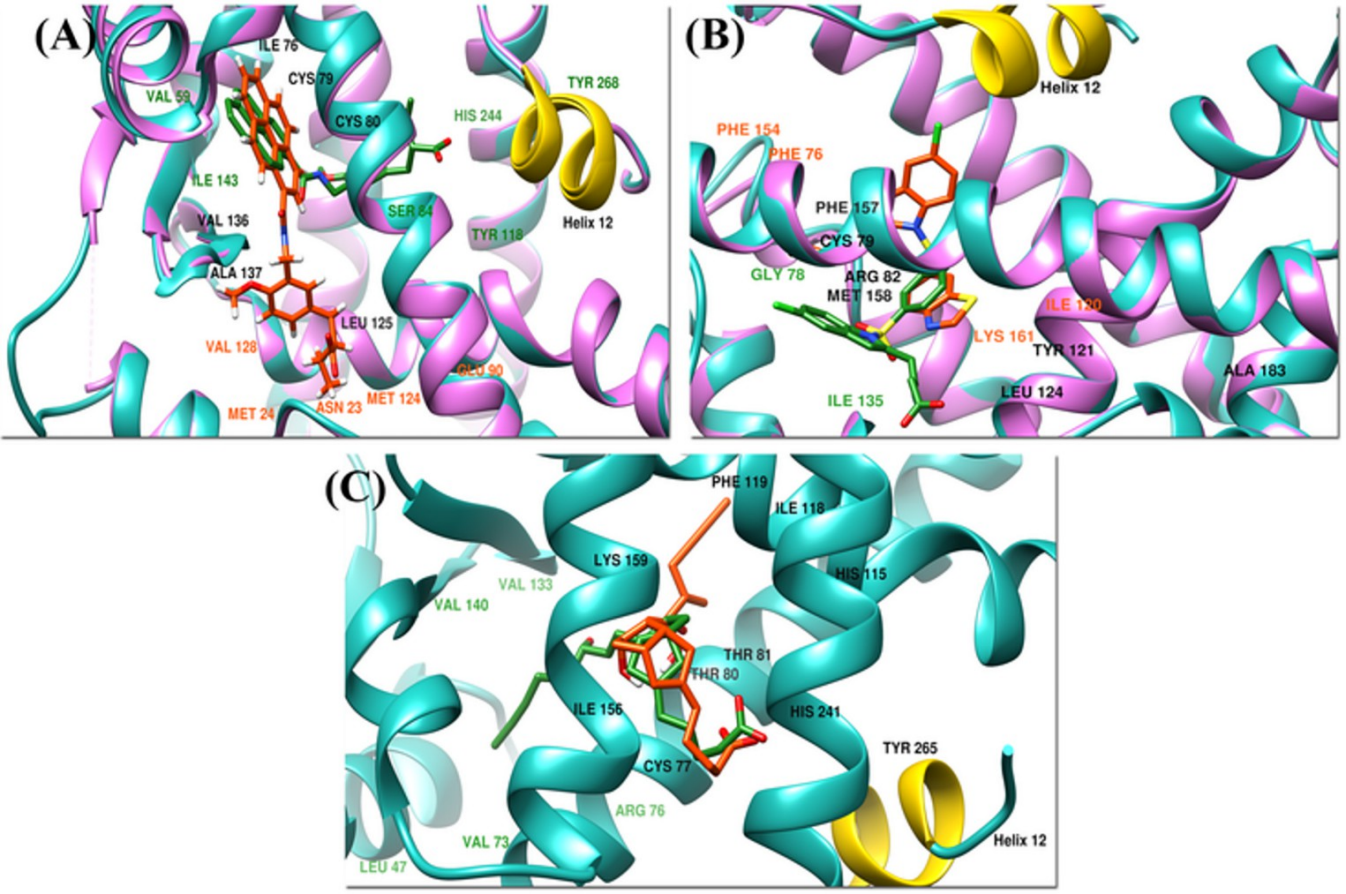
The superimposed crystallographic pose (receptor in light sea green and ligand in dark green) and docked pose (receptor in hot pink and ligand in orange) of (A) PPARα, (B) PPARγ and (C) PPARδ. Helix-12 is represented in yellow.

The performance of the docking algorithm is evaluated by redocking the ligands using the conformations discovered in the X-ray structures. The crystal ligands APHM13, Lanifibranor, and Iloprost successfully docked back to PPARα, PPARγ, and PPARδ in their binding pockets showing the LibDockScore of 155.849, 104.357, and 128.873, respectively. The RMSD between docked pose and crystallographic pose of APHM13:PPARα and Lanifibranor: PPARγ were 0.477 Å and 0.392 Å, respectively, due to a subtle difference in the binding residues but for Iloprost: PPARδ RMSD of 0.00 Å was observed. Upon 2D interaction analysis using DS, it was revealed that residues Ile76, Cys79, Cys80, Leu125, Val136, and Ala137 of PPARα were the common residues forming interaction with APHM13 in both docked complex and crystallographic complex. In addition to this, APHM13 also contacted with PPARα other residues: Val59, Ser84, Ile143, Tyr118, His244, and Tyr268 in crystallographic complex whereas in docked complex the additional residues that showed interaction were: Asn23, Met24, Glu90, Met124, and Val128of PPARα. The 3D interaction diagram of APHM13 in crystal pose and docked pose is illustrated in Fig 8(A).

The Lanifibranor showed interaction with residues of PPARγ: Cys79, Arg82, Tyr121, Leu124, Phe157, Met158, and Ser183 in both complexes. Moreover, some residues Phe76, Ile120, Phe154, and Lys161 of PPARγ were also involved in interaction in docked complex instead of residues Gly78 and Ile135 which were engaged in interaction with Lanifibranor in the crystallographic complex (Fig 8(B)).

In docked complex, the interaction between Iloprost and PPARδ included the involvement of residues Cys77, Thr80, Thr81, His115, Ile118, Phe119, leu122, Ile156, Lys159, His241, and Tyr265 which were all also making interactions in crystal complex although some additional residues Leu47, Val73, Cys76, Val133, and Val140 were also observed interacting with Iloprost in crystal complex (Fig 8(C)).

#### 3.4.2. FDA-approved drugs library docking

The highest LibDock score for PPARα was 119.43 shown by Forasartan. The key residues for binding interaction between Forasartan and PPARα were Met24, Cys80, Thr87, Ile121, Leu125, and Val128. Thr87 formed a carbon-hydrogen bond whereas Ile12, Met24, Cys80, Leu125, and Val128 were forming hydrophobic interactions with Forasartan. It is used as an antihypertensive agent to treat hypertension and also is a selective angiotensin II antagonist, type 1; because angiotensin induces vasoconstriction, inhibiting this receptor reduces vasoconstriction, which consequently also decreases vascular resistance [67]. Forasartan has a high affinity for the AT1 receptor (IC50 = 2.9 +/- 0.1nM) [68].

**Fig 9:**
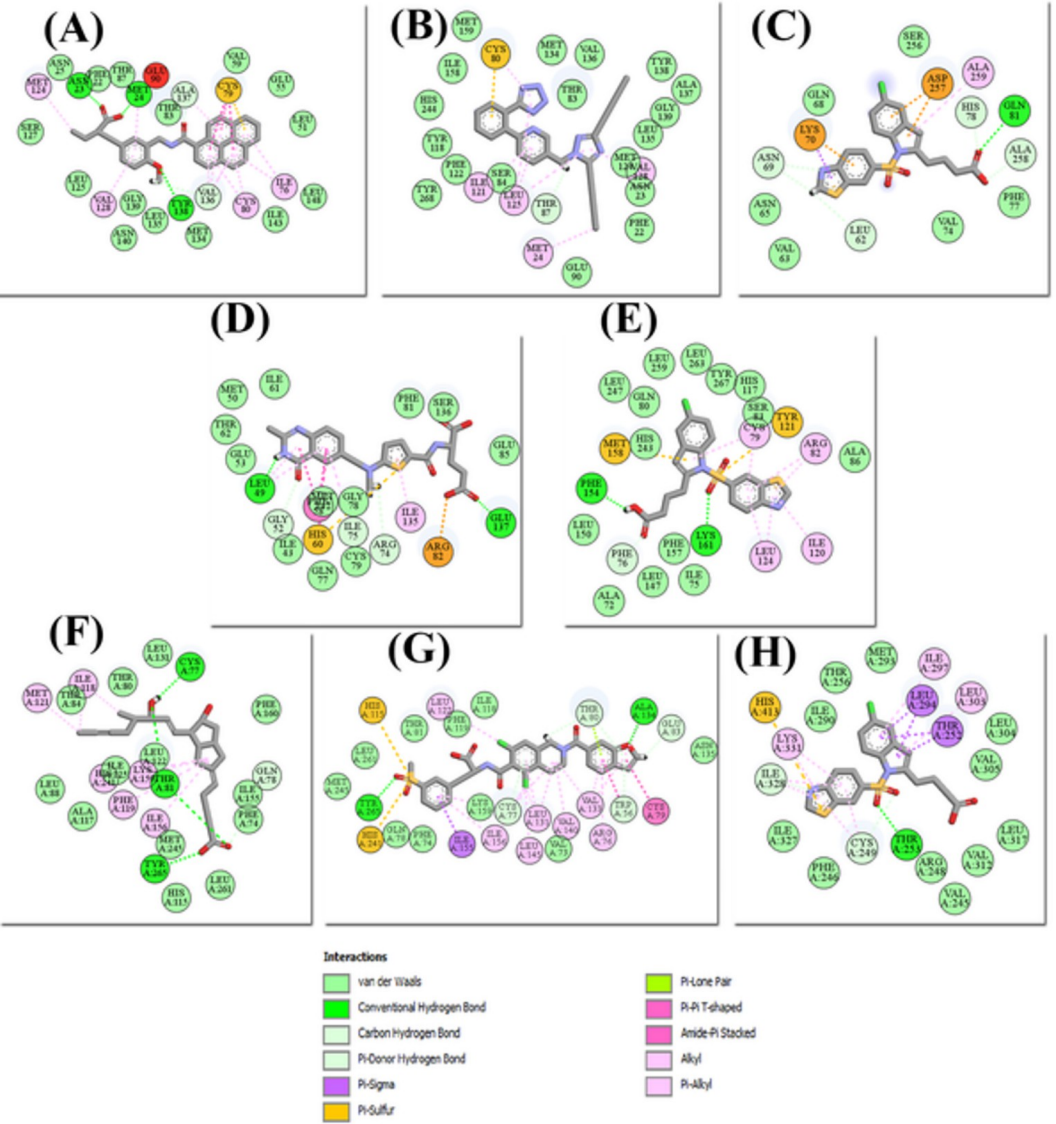
The 2D interaction diagrams of (A) APHM13 with PPARα, (B) Forasartan with PPARα, (C) Lanifibranor with PPARα, (D) Raltitrexed with PPARγ, (E) Lanifibranor with PPARγ, (F) Iloprost with PPARδ, (G) Lifitegrast with PPARδ and (H) Lanifibranor with PPARδ sketched through Discovery Studio.

The highest docked FDA-approved drug for PPAR was Raltitrexed, which had a LibDock score of 151.057. The binding interaction of Raltitrexed with residues: Leu49, Gly52, Phe58, His60, Arg82, Ile135, and Glu137 of PPARγ were noted. The residues of PPARγ: Gly52, Arg74, and Glu137 were developing carbon-hydrogen bonds and conventional hydrogen bonds whereas the other residues Gly52, Phe58, His60, Arg82, and Glu137 were forming hydrophobic interactions with Raltitrexed. Leu49 and Ile75 were involved in both types of interaction. It is a thymidylate synthase (TS) inhibitor that inhibits L1210 cell growth that belongs to the antimetabolite class of cytotoxic medicines (IC50 = nM) [69]. It is also effective as a single agent in colorectal cancer (CRC) and is often given with other cancer drugs [70]. The binding mode of Forasartan, Raltitrexed, and Lifitegrast is shown in Fig 9(B), 9(D) and 9(G).

The top docked FDA-approved drug for PPARδ was Lifitegrast. It showed the LibDock score of 162.505. Two hydrogen bonds were established during docking of Lifitegrast with PPARδ. The PPARδ residues Trp228, Cys249, Thr252, Glu255, Ala306, and Tyr437 formed hydrogen bonds. All other residues such as Trp228, Arg248, Leu249, Cys251, His287, Leu303, Val305, Val312, Ile327, Ile328, and His413 were involved in hydrophobic contacts. Lifitegrast is an antagonist that inhibits the T cell-mediated inflammatory cycle by acting as a direct competitive antagonist of ICAM-1 to LFA-1 binding [71]. In vitro, Lifitegrast inhibited Jurkat T cell adherence to ICAM-1 in a concentration-dependent manner (IC50 = 2.98 nmol/L) [72, 73]. It is used to treat keratoconjunctivitis sicca also known as dry eye syndrome (DED). It also functions as an anti-inflammatory agent as well as a lymphocyte function-associated antigen-1 antagonist [74, 75]. The three top scored hits Forasartan for PPARα, Raltitrexed for PPARγ, and Lifitegrast for PPARδ were further selected for simulation studies according to their respective subtype, as they were filtered out during pharmacophore screening for the other two subtypes.

#### 3.4.3. Lanifibranor docking

The LibDock score of Lanifibranor with PPARα and PPARδ were 92.648 and 112.648, respectively. The 2D interaction analysis of the Lanifibranor-PPARα complex revealed that the residues leu62, Asn69, His78, and Ala258 made carbon-hydrogen bonds whereas Gln81 formed a conventional hydrogen bond with Lanifibranor. Lys70, Asp257, and Ala259 were observed in hydrophobic contacts. The 2D interaction diagrams of Lanifibranor with all PPARs are shown in Fig 9(C), 9(E) and 9(G). Upon docking, the Lanifibranor formed extensive hydrophobic interactions with PPARδ involving residues Thr252, Leu294, Ile297, Ile328, Lys331, and His413. Thr253 was the only residue that contributed to conventional hydrogen bond formation. Lanifibranor engaged Cys249 and Ile328 in both carbon-hydrogen bond and hydrophobic interactions.

### 3.5. Fingerprinting

Tanimoto coefficient (Tc) was used to evaluate the structural similarity between molecules using overlapping molecular fingerprints. The compound having minimum similarity with Lanifibranor was Raltitrexed, having a similarity score of 0.575099 and the compound with maximum similarity was Lifitegrast with a 66% score that is 0.669944 whereas Forasartan showed the similarity score of 60%. All three compounds showed similarity >50% with Lanifibranor.

On similarity search, it was found that methylsulfonyl benzene, chlorobenzene, and the butyric acid group were present in both Lifitegrast and Lanifibranor. The Lanifibranor and Raltitrexed had a butyric acid group common in both. These groups can be effective in designing new drugs for NASH. Furthermore, it was also observed that the usual structural features of PPAR agonists such as sulfur, carbon, oxygen, and nitrogen except fluoride were also present in these compounds [66]. The detail of the similarity search is listed in Table 4.

**Table 4:** The similarity score obtained from fingerprinting.

Compounds	Similarity	SA	SB	SC
**Lifitegrast**	0.669944	477	303	-68
**Forasartan**	0.6098	336	142	73
**Raltitrexed**	0.575099	291	97	118

### 3.6. Molecular dynamics simulations

The molecular dynamic method is frequently used to analyze atom behavior, structural stability, and atomic-level conformational changes. Molecular dynamic simulations are the most prominent tool used in the all-atom modeling of biomolecules to get insight into the system’s dynamical characteristics. Through MD simulations, the protein interactions, conformational, and structural modification in the protein, and the ligand associated movements within the hydrated environment can be studied.

The MD simulation of 200 nanoseconds (ns) was carried out for each complex to measure the behavior of docked complexes in real-time. The MD simulation trajectories were evaluated by the time series of root mean square deviation (RMSD) and root mean square fluctuation (RMSF). Upon ligand binding, the protein in all PPAR complexes does not undergo significant structural changes. However, it is generally known that PPAR has a significant degree of flexibility, particularly in the Ω-loop. This loop is highly disordered and has high molecular flexibility, due to which it remains unmodeled in many PPAR X-ray crystal structures [76–78].

#### 3.6.1. PPARα complexes

**Fig 10:**
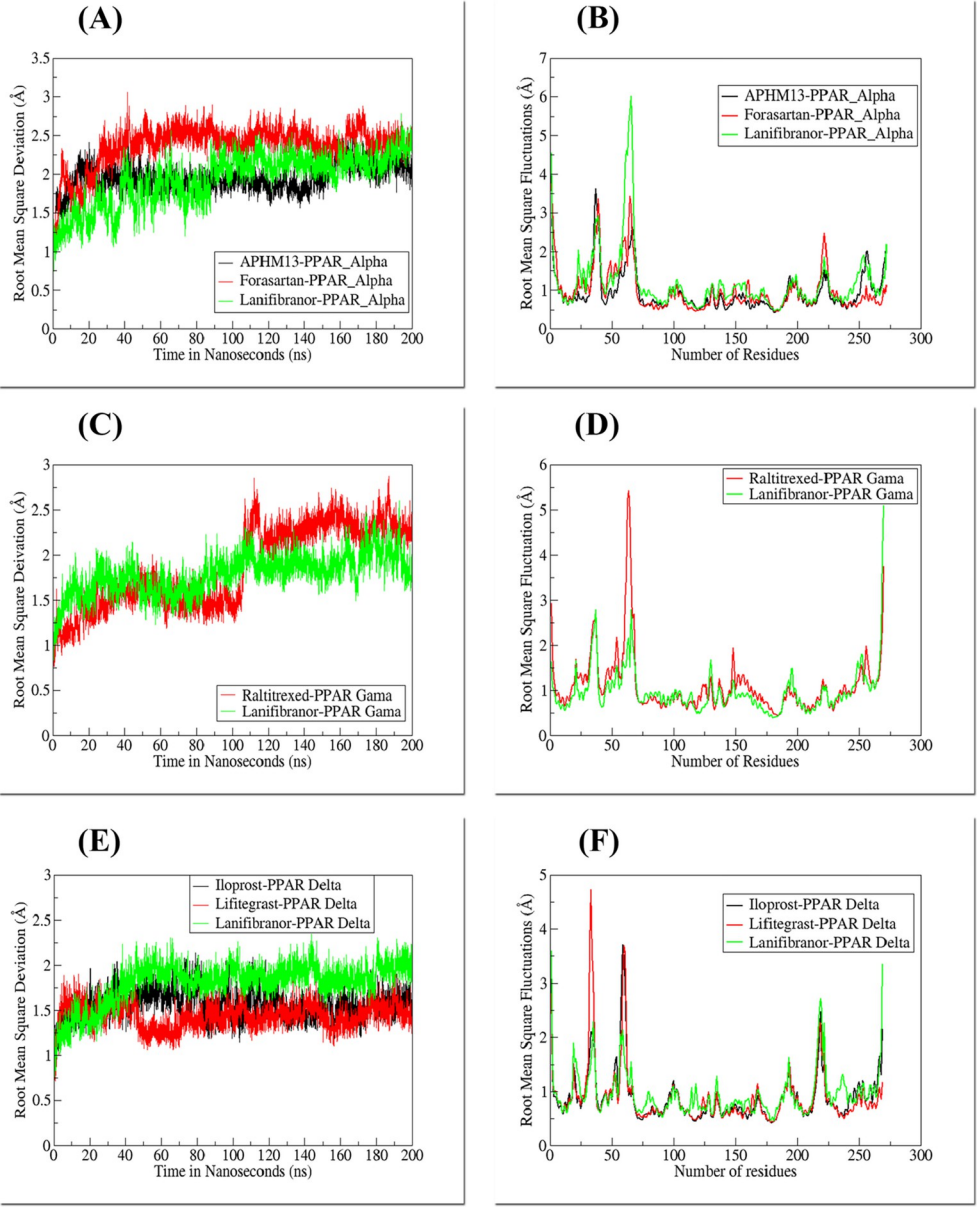
RSMD and RMSF plots of all complexes.

**Fig 11:**
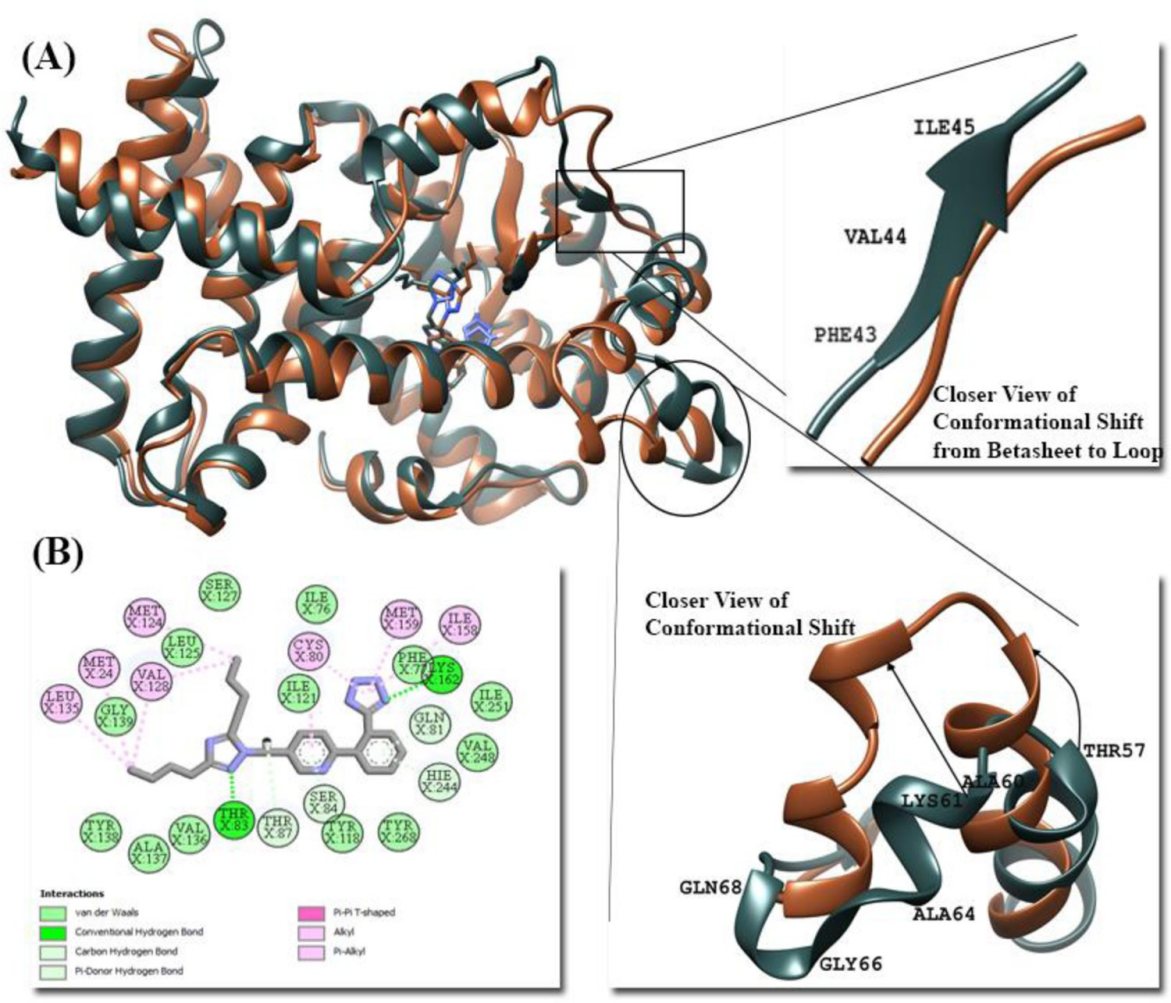
(A) Superimposed structure of pdbs of PPARα-Forasartan complex at 0-ns (dark slate grey) and 200-ns (Sienna) and closer view of conformational shifts. (B) 2D interaction of Forasartan-PPARα at 200-ns.

The mean RMSD of crystal bound ligand APHM13, FDA approved drug Forasartan and clinical trial Pan-PPAR drug Lanifibranor were 1.94 Å, 2.36 Å, and 1.92 Å, respectively. The combined trajectories of all PPARα complexes during the 200-ns time are illustrated in Fig 10(A) and 10(B). Initially, the RMSD of the APHM13-PPARα complex increased from zero to 20 ns, with the mean of 1.7 Å, later from 20 ns to 153 ns the equilibrium was observed in RMSD through the trajectory plot followed by a gradual increase showing the highest RMSD at 176 ns of 2.52 Å after that the complex attained stability. The ligand showed no movement and was continuously interacting with the residues of the binding site. In the Forasartan-PPARα complex, an abrupt change in RMSD was observed from zero ns to 5 ns, followed by a decrease in RMSD to 15 ns. The former shift was explored as a complex’s sudden exposure adjustment to a dynamic environment. Afterward, the gradual increase in RMSD value occurred, showing the highest peak at 41 ns of 3.05 Å. During simulation, Forasartan’s orientational shift allowed it to engage with two key residues: Ser84 and His244, both of which are required for agonist-induced PPAR activation [79]. The superimposed structure of pdbs of 0-ns and 200-ns scale can be seen in Fig 11.

**Fig 12:**
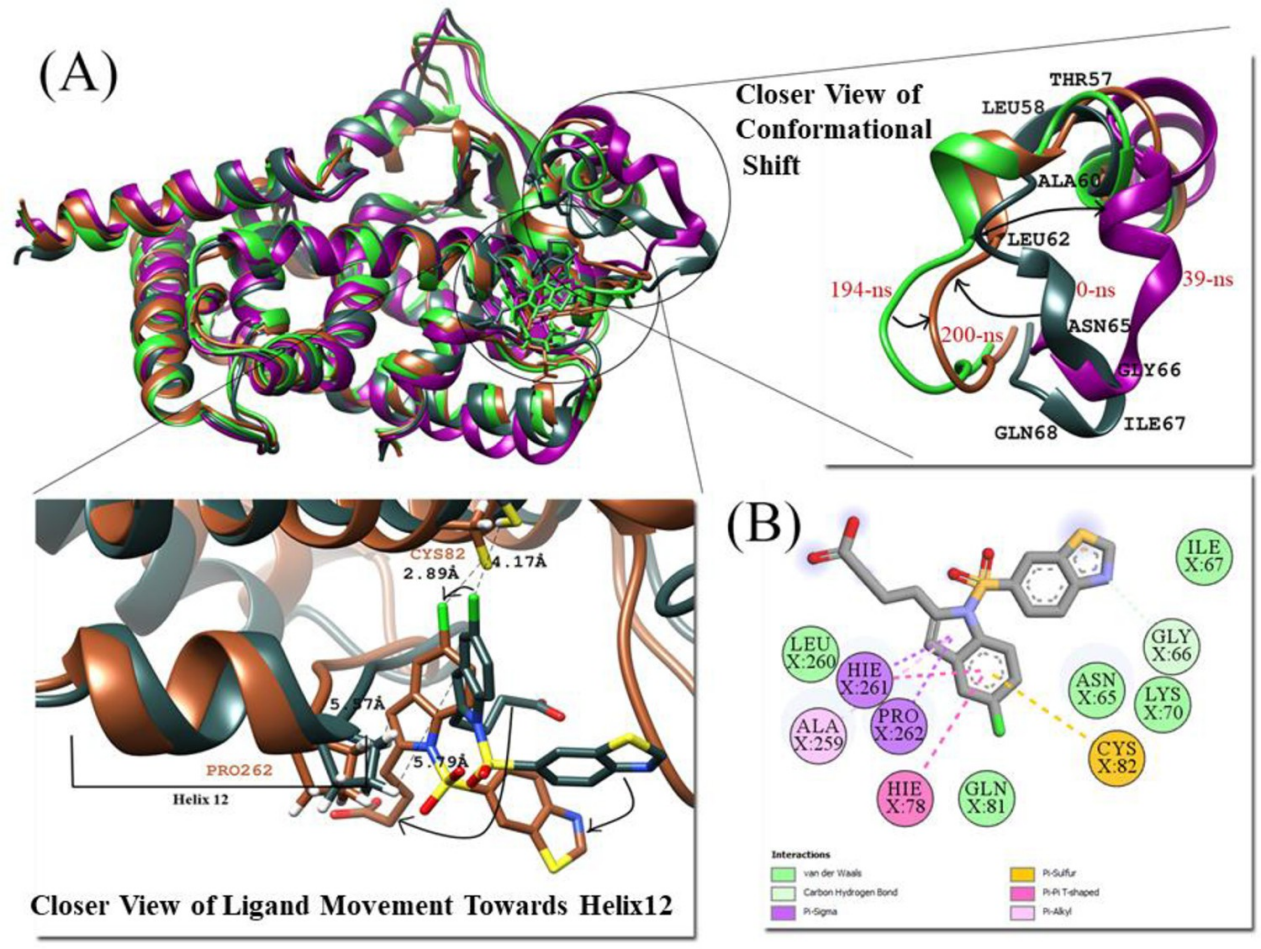
(A) Superimposed structure of pdbs of PPARα-Lanifibranor complex at 0-ns (dark slate grey), 39-ns (dark magenta), 194-ns (green), and 200-ns (Sienna). A closer view of conformational changes occurred in PPARα during simulation and Movement of Lanifibranor towards H-12. (B) 2D interaction of Lanifibranor-PPARα at 200-ns.

Among all PPARα complexes, more fluctuations were observed in the Lanifibranor-PPARα complex. From zero ns to 87 ns, the fluctuations and random peaks were observed but afterward, the plot showed fewer fluctuations and more stability towards the very end of the simulation. The Lanifibranor-PPARα RSMD plot showed the highest peak at 192 ns of 2.78 Å. The variability in the RMSD of Lanifibranor-PPARα was detected due to the noteworthy movement of Lanifibranor in the docked pocket and inward movement of Ω-loop which brought Lanifibranor closer to the Helix-12 (H-12) of PPARα and developed new interactions with the residues (Pro262 and Gln265) of H-12 (Fig 12).

The RMSF was evaluated for residue-by-residue fluctuation of docked protein during simulation. The RMSF average value for APHM13, Forasartan, and Lanifibranor complex was 0.94 Å, 0.98 Å, and 1.18 Å, respectively. Among all PPARα complexes, the highest RMSF average value was observed for the Lanifibranor-PPARα complex as for the RMSD value, which indicates greater flexibility in the residues during simulation (Fig 12). In all complexes, the fluctuations were observed in the loop between helix-2 and beta-sheet-1 (Ser34-Pro42), Ω-loop (Ala64-Glu71), and helix11-helix12 loop at C-terminus (Thr254 and His261). The same secondary structure shift was observed in PPARα-APHM13 and PPARα-Forasartan complexes, the change was of beta-sheet (Phe43-Ile45) conversion to loop near the active pocket. Whereas away from the binding pocket, the interconversion of the helix near Ω-loop (Ala60-Val63) to loop and loop to helix occurred was observed in all PPARα complexes at different times during the simulation. The Ω-loop in Lanifibranor-PPARα complex showed more fluctuations than in APHM13-PPARα and Forasartan-PPARα complexes. The fluctuating residues of Ω-loop were the most liable area for the uneven RMSF. The last set of residues was of C-terminus residues that showed little mobility.

#### 3.6.2. PPARγ complexes

**Fig 13:**
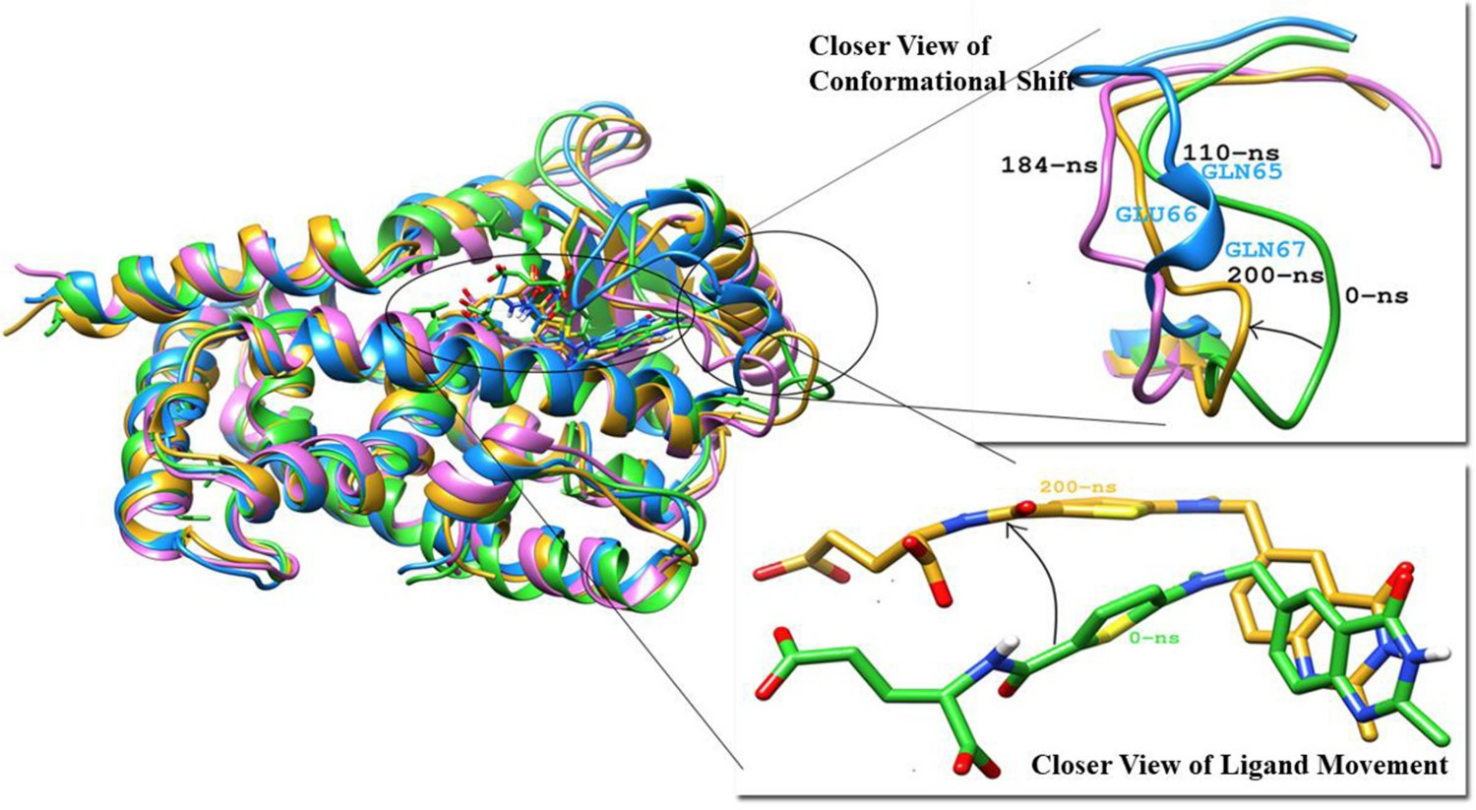
(A) Superimposed structures of pdb of Raltitrexed-PPARγ Complex at 0-ns (lime green), 110-ns (dodger blue), 184-ns (orchid), and 200-ns (gold), and closer view of conformational shift and Raltitrexed movement.

To measure the deviation and conformation stability of backbone atoms the RMSD of all PPARγ complexes were computed. Average RMSD of protein elucidates the stability of the structure and it is also an important indicator of the biological process. The mean RMSD value of Raltitrexed-PPARγ and Lanifibranor-PPARγ complexes were 1.8 Å and 1.7 Å respectively (Fig 10(C) and 10(D)). A rise in RMSD was observed at 110 ns, which was due to the formation of a small helix at residues (Gln65, Glu66, and Gln67) in the Ω-loop (Fig 13) which again attained its original structure that is the loop. Raltitrexed’s movement was witnessed in the docked pocket, but it didn’t cause any major change in interacting residues. The 2-methylquinazolin-4(3H)-one ring of the ligand was more stable and little movement occurred at its place while the rest part ((S)-2-(5-(dimethylamino)thiophene-2-carboxamido)pentanedioic acid) of the Raltitrexed was more flexible and changed its position in the docking pocket at various time of the simulation (Fig 13).

**Fig 14:**
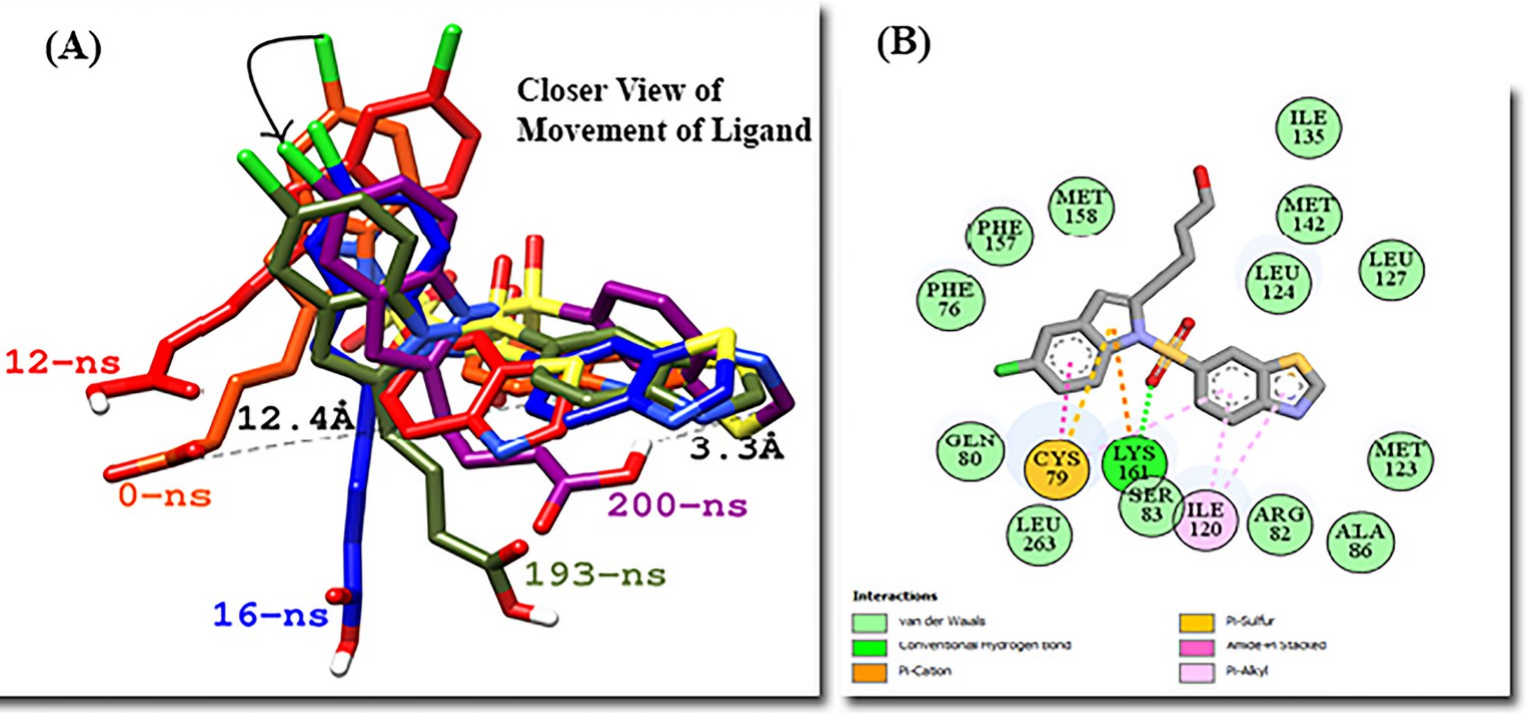
(A) Movement of Lanifibranor in PPARγ at different time scales. (B) 2D interaction diagram of Lanifibranor at 200ns with interacting residues of PPARγ.

The Lanifibranor-PPARγ complex shows the stable RMSD plot. The pentanoic acid group of Lanifibranor constantly changed its interacting residues. At the beginning of the simulation, the 5-chloro-1H-indole group was interacting with Gln80, His243, Leu247, and Leu263 and the pentanoic acid was interacting with Phe76 and Phe154. At 80ns, Cys79, Phe76, and His243 engaged with the 5-chloro-1H-indole group, whereas Phe154 formed a hydrogen bond with pentanoic acid. During simulation, the ligand structure compacted, bringing the pentanoic acid and benzo[d]thiazole rings closer. At the end of the simulation, residues Cys79 were interacting with both the 5-chloro-1H-indole group and the benzo[d]thiazole group. Lys161 made contact with the 5-chloro-1H-indole group and sulfur dioxide which linked both these groups. Ile120 was seen interacting with the benzo[d]thiazole group whereas no residue was seen interacting with pentanoic acid (Fig 14).

The majority of subtle conformational changes in PPARγ complexes were seen within the loop (Gly33-Asp45) and Ω-loop (Lys55-Glu70). The fluctuations in H-12 were detected during trajectory analysis. This might be due to the ligand’s lack of direct interaction with H-12. The mean RMSF of 1.10 Å and 0.97 Å were calculated for Raltitrexed-PPARγ and Lanifibranor-PPARγ, respectively. The ligands were interacting with the residues of Arm II and Arm III. Both ligands bind to PPARγ and partially activate it.

#### 3.6.3. PPARδ complexes

RMSD analysis was performed to measure the similarity between two superimposed atomic coordinates. The RMSD analysis for PPARδ complexes showed the mean RMSD for crystal bound ligand Iloprost, FDA-approved drug Lifitegrast and Lanifibranor 1.56 Å, 1.44 Å, and 1.80 Å, respectively (Fig 10(E) and 10(F)). The RMSD plot of all PPARδ complexes showed tremendous stability. Trajectory analysis revealed that all three PPARδ ligands were well anchored and showed interaction with the residues of the binding site throughout the simulation. The majority of fluctuations were detected in the loop regions. The fluctuations were associated with the structural stability and movements during MD simulations. The general pattern of RMSD in Iloprost-PPARδ and Lifitegrast-PPARδ systems did not reveal any noteworthy structural fluctuations or structural shifts, indicating the complex’s stability. The Iloprost and Lifitegrast stayed at their place throughout the simulation whereas the abrupt exposure adjustment of the Lanifibranor-PPARδ complex to a dynamic environment resulted in the rise in RMSD value in the beginning. The Lanifibranor changed its orientation, and a notable movement of the 5-chloro-1H-indole group was observed at 50 ns. After that, significant conformational changes were observed. The change in orientation helped the ligand to form stronger interaction with PPARδ. The His413 established a hydrogen bond with the ligand’s thiazol group.

The root-mean-square fluctuation (RMSF) measures the average fluctuations of protein residue over the time course. It measures the deviations of protein residues from a reference position. The mean RMSF for PPARδ in complex with Iloprost and Lifitegrast were same i.e., 0.88 Å whereas the mean RMSF of 0.94 Å was observed for Lanifibranor-PPARδ (Fig 10(E) and 10(F)). The fluctuations occur in the protein residues to make the ligand well anchored and stable at its place. The significant fluctuations were observed in loops: entrance loop of the cavity (Gly303-Pro210), Ω-loop (Gly225-Lys239), and loop (His389-Tyr394) in all three complexes. In Iloprost-PPARδ, the maximum fluctuation of 3.7 Å was shown at Gln230. During 150 ns, a small helix was formed by Gln230-Asn233, after that it disappeared and never formed again till the end of the simulation and the extension and contraction of H-12 was also observed throughout the simulation.

**Fig 15:**
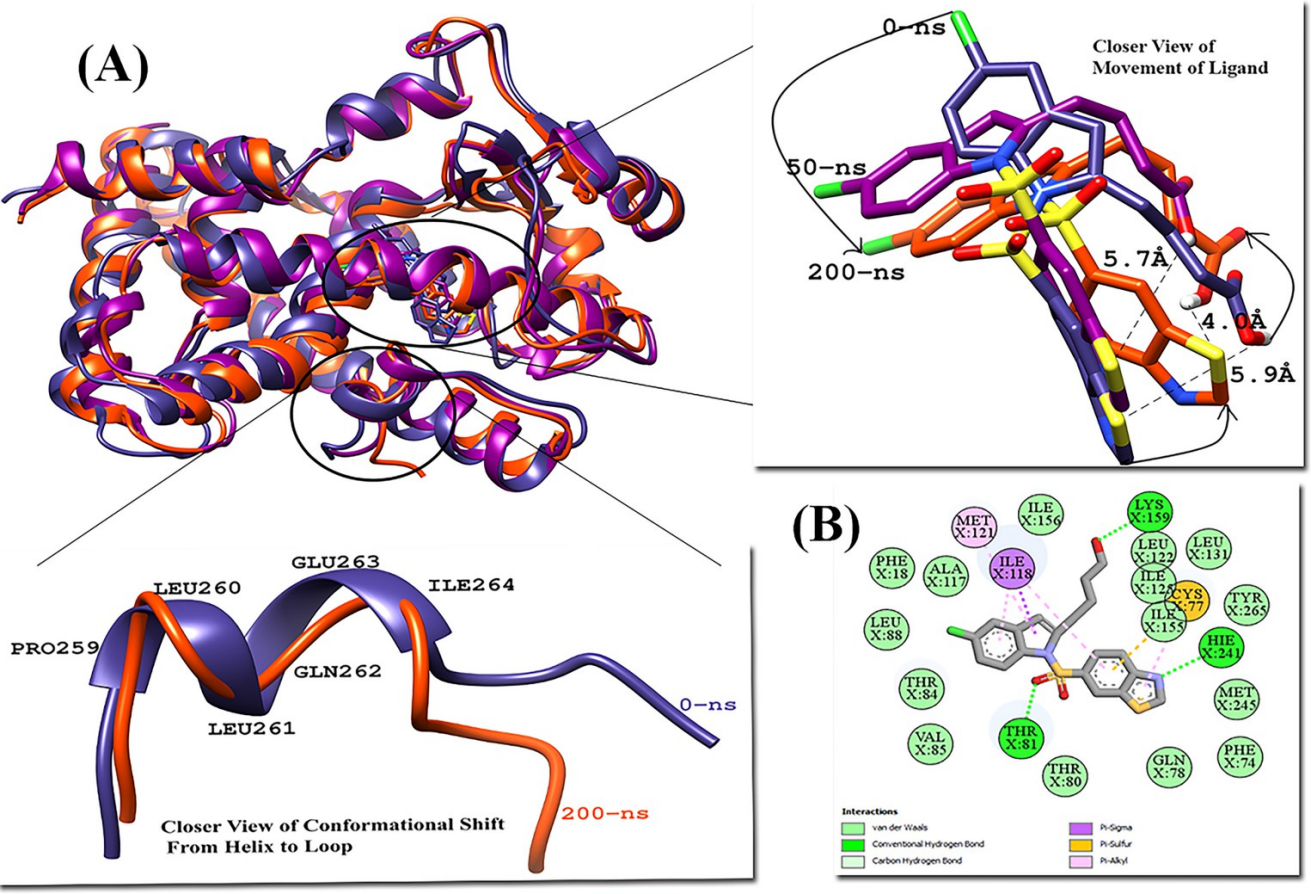
(A) Superimposed structures of pdb of PPARδ-Lanifibranor Complex, Movement of ligand at different time scales during the simulation, and Closer view of conformational changes at H-12. (B) 2D interaction of Lanifibranor with PPARδ at 50-ns time.

In Lifitegrast-PPARδ, the maximum fluctuation of 4.7 Å was shown by residue Lys204, away from the binding pocket. This residue is present at the entrance to the ligand-binding site, it might be crucial in making the ligand intact with the protein by providing flexibility to the overall structure. Other than these sets of residues, the residues of the active site (Ala306-Gly308) were structurally changing from loop to helix and helix to loop during simulation. Asn307 is the most flexible residue it allows the large ligand to enter the ligand-binding cavity [80]. In Lanifibranor-PPARδ another structural change was observed at 190 ns in the important residues, the H-12 changed into a loop but at 200 ns it again adopted its original structure (Fig 15). The helical structures of activation function-2 (AF-2) provide hydrophobic docking sites for Nuclear Receptor coactivators and are crucial for the ligand-dependent transactivation activity of Nuclear receptors [81]. All three PPARδ ligands were forming bonds with amino acid residues Thr253, His287, His413, and Tyr437 which are crucial for stabilizing the AF-2 domain [79].

### 3.7. Hydrogen bond analysis

The formation and breaking of hydrogen bonds are reported to be an essential discipline in the development of protein stability and flexibility.

#### 3.7.1. Hydrogen bond analysis of PPARα complexes

**Fig 16:**
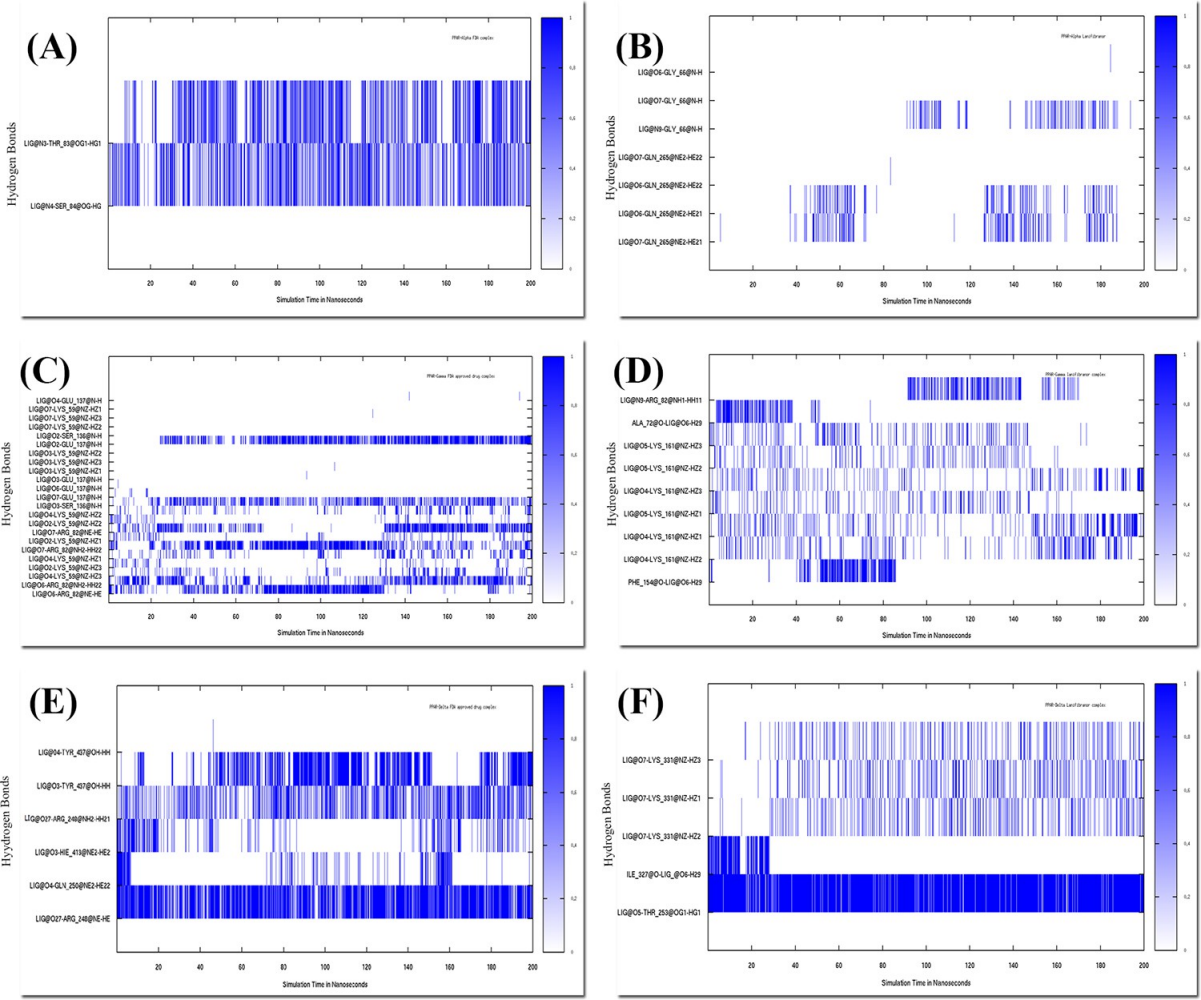
Hydrogen bond analysis of (A) PPARα-Forasartan, (B) PPARα-Lanifibranor, (C) PPARγ-Raltitrexed, (D) PPARγ-Lanifibranor, (E) PPARδ-Lifitegrast, (F)PPARδ-Lanifibranor.

In APHM13-PPARα several residues were participating in hydrogen bonds and contributing to the stability. The residues of PPARα Asn23, Met24, Thr83, Thr87, Ala137, and Thr138 were involved in making hydrogen bonding with APHM13. All these residues were making more than one hydrogen bond except Thr83. Asn23, Met24, and Thr87 formed hydrogen bonds with O2 and O4 of the ligand. These bonds were forming and interchanging with one another during the simulation. Asn23 and Met24 were not seen forming bonds after 160 ns and 150 ns respectively while can also be seen forming hydrogen bonds at 200ns with O2 of the APHM13. The OH of Tyr138 was forming the hydrogen bond with O2 and O4, and O35 of the ligand was interacting with N of Tyr138. Tyr138 with O35 was making a strong and consistent bond till 180 ns after that the bond was not observed in the plot. Forasartan interacting with PPARα formed two hydrogen bonds (Fig 16(A)). Thr83 and Ser84 formed Hydrogen bonds with N3 and N4 respectively, of Forasartan. These bonds were strong and consistent bonds that played an important role in keeping Forasartan and PPARα in contact and stable throughout the simulation. In Lanifibranor-PPARα, Gly66 and Gn265 were involved in hydrogen bonds (Fig 16(B)). At the beginning of the simulation, no hydrogen was displayed, at 40 ns Gln265 formed a bond with O6 and O7 of Lanifibranor whereas Gly99 formed a hydrogen bond at 90 ns. The appearance and disappearance of the bonds were due to the movement of the ligand in the binding cavity.

#### 3.7.2. Hydrogen bond analysis of PPARγ complexes

Numerous hydrogen bonds were found throughout the simulation of the Raltitrexed-PPARγ complex. Residues of PPARγ: Lys59, Arg82, Ser136, and Glu137 were involved in strong hydrogen bonding and were consistent while other residues forming hydrogen bonds were seen on and off throughout the simulation with Raltitrexed (Fig 16(C)). NZ atom of Lys59 was forming hydrogen bonds with the O2, O3, O4, and O7. The fluctuating residue Lys59 of Ω-loop was making a hydrogen bond with various atoms of the Raltitrexed such as O2, O3, O4, and O7. The bond with O2 atom and O4 atom of ligand was more coherent so their radial distribution was analyzed using RDF analysis. NE atom and NH2 atom of Arg82 were alternatively involved in hydrogen bond formation with O6. The O6 atom was forming a bond throughout the simulation course. The ligand’s O3 and O2 atoms were establishing hydrogen bonds with Ser136 and Glu137. Glu137 was not involved in bond formation at the start of the simulation, but it began to make bonds at 25 ns and remained visible until the completion of the simulation, suggesting that it aids in maintaining the ligands in place. During the simulation of Lanifibranor-PPARγ, the residues of PPARγ: Ala72, Arg82, Phe154, and Lys161 were participating in hydrogen bond formation (Fig 16(D)). By 40 ns of simulation, the O6 was creating a stronger hydrogen bond with Phe154, but after 85 ns, the O6 was no longer visible forming a hydrogen bond, which was owing to the ligand’s change in conformation. Whereas Lys131 was found forming two hydrogen bonds i.e: with O4 and O5 of the Lanifibranor throughout the simulation trajectories, as a result, the ligand mobility within the binding cavity was detected.

#### 3.7.3. Hydrogen bond analysis of PPARδ complexes

For more than 80% of the simulation time, hydrogen bonding was detected in PPARδ complexes. PPARδ in complex with crystal ligand, Hydrogen bonds formed at the docking, Cys249, and Thr437 were diminished during the simulation while Thr253 was forming strong bond alternately with O6 and O8 of the ligand, throughout the simulation duration. New hydrogen bonds formed between Gln250, Thr256, His287 His413, and ligand atoms. Bonds between time duration of 100 ns to 160 ns were not observed for Gln250 and Thr256, due to change in orientation of the ligand. The formation and breaking of the hydrogen bonds at various time was noted. Hydrogen bonds in PPARδ and Lifitegrast complex involved, Arg248, Gln250, His413, and Tyr437 reside with the ligand. Gln250 forming a hydrogen bond with the O4 of the ligand was not observed after 160 ns (Fig 16(E)). Arg248, His413, and Tyr437 help in the binding of ligand during 200 ns simulation. NE and NH2 of Arg248 were forming bonds simultaneously with the O27 of the ligand. These residues play a vital role in keeping the ligand in a particular orientation. Thr253 and Lys331 were the strongest hydrogen bonds in the Lanifibranor-PPARδ complex (Fig 16(F)). Thr253 was showing hydrogen bond interaction with the O5 of the ligand whereas, in case of Lys331, NZ-HZ1, NZ-HZ2, and NZ-HZ3 atoms were making hydrogen bonds with the O7 of the ligand interchangeably. In the beginning, Ile155 was observed forming a bond with the O6 of the ligand but then it varnished due to a movement in the ring of Lanifibranor. Thr253 and Lys331 were participating in constant and strong hydrogen bond interactions that stabilized the ligand within the cavity. The trajectory analysis showed the presence of stable hydrogen bonding among the complexes.

### 3.8. Radial distribution function

Important residues of the binding site that stabilized the agonist throughout the simulation were imperiled to the RDF [53] to perform their role in agonist binding. It has been employed to determine the distribution of atoms and molecules surrounding targets.

#### 3.8.1. RDF of PPARα complexes

The APHM13-PPAR complex included many hydrogen bonds but the Thr138: N was making the most consistent hydrogen bond interaction with the O35 atom of the ligand. Initially, the distribution peak was not sharp and exhibited the g(r) value of 0.61 at 3.01 Å whereas, at 100 ns, the sharp definite peak was observed having the g(r) value of 1.61 at 2.92 Å. After simulation, the highest distribution was observed at 4.9 Å with the g(r) value of 0.24. The peak was seen to be high in the middle of the simulation, and the density distribution expanded enhances the likelihood of the probability of finding ligand around Thr135. The lowering of density and increase in the distance occurred at the end simulation because of the involvement of the O35 atom of the ligand with another residue; Ala137.

**Fig 17:**
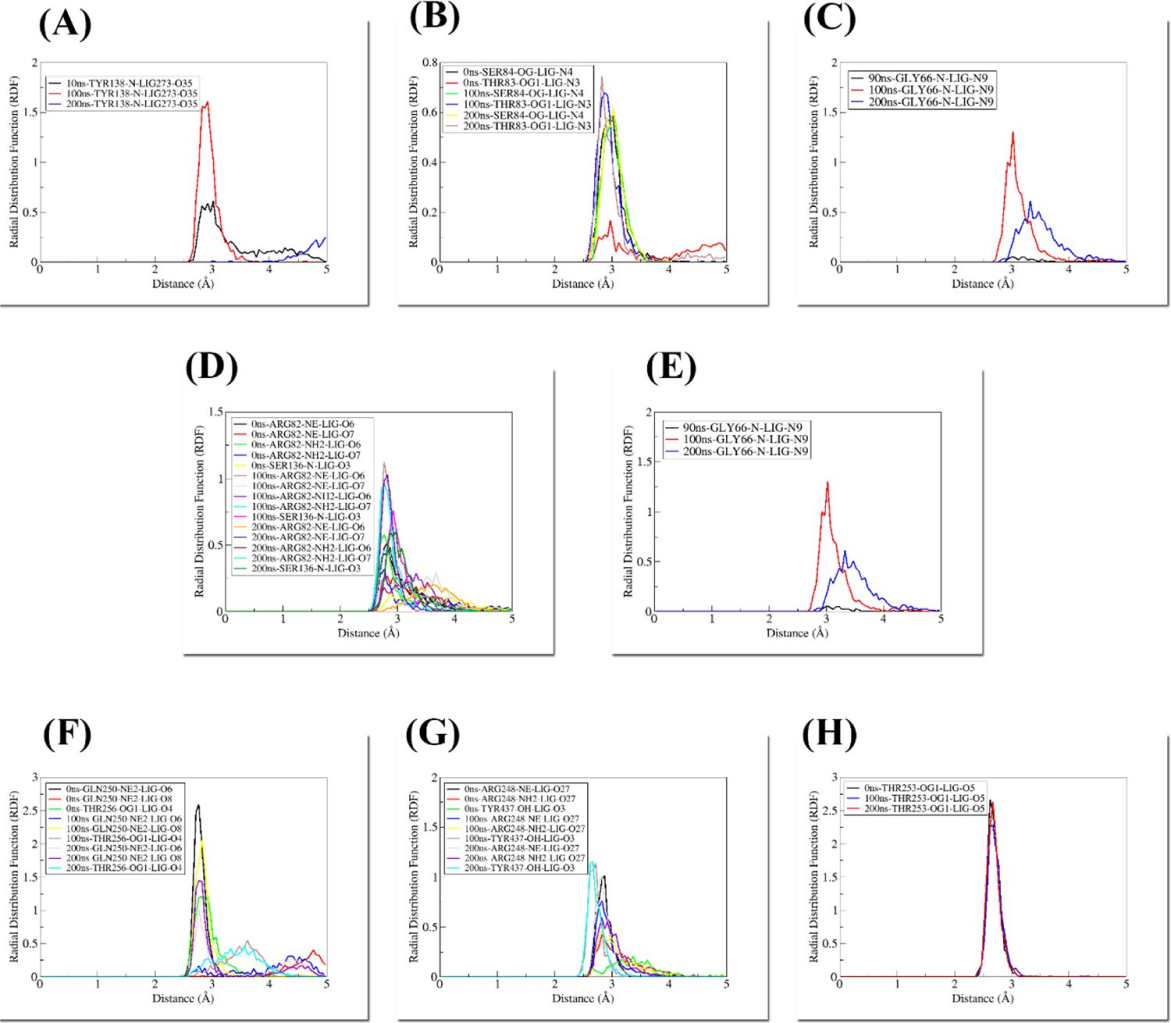
Radial Distribution Function calculated for (A) PPARα-APHM13, (B) PPARα-Forasartan, (C) PPARα-Lanifibranor, (D) PPARγ-Raltitrexed, (E) PPARγ-Lanifibranor, (F) PPARδ-Iloprost, (G) PPARδ-Lifitegrast, (H) PPARδ-Lanifibranor.

The active site residues of PPARα: Thr83 and Ser84 were found as vital residues involved in Hydrogen bond interaction with Forasartan (Fig 16(A)). Initially, the distribution peak for Thr83:OG1 and N3 atom of Forasartan was observed having g(r) of 0.16 at the distance of 2.97 Å. At 100 ns of simulation, the peak was observed at a distance 0.67 Å showing a g(r) value 2.87. Finally, towards the end of the simulation, this distribution revealed a g(r) value of 0.62 at 3.03 Å. The RDF analysis for Ser84:OG showed that at the start and in the middle of simulation duration the distribution was almost the same such that 0.58 and 0.60 at the same distance 3.02 Å while the sharp and a definite peak was observed at the end of the simulation with the increase in g(r) of 0.74 and decrease in distance of 2.8 Å (Fig 17(A)). This shows that the ligand moved closer to the binding site and interaction became stronger.

The RDF of Gly66:N of PPARα and N9 atom of Lanifibranor was performed (Fig 17(B)). Due to the mobility of the Lanifibranor, no hydrogen bond was seen in the PPARα-Lanifibranor complex at the beginning of the simulation. The sharp peak was detected at 100 ns with the value of g(r) 1.31 at 3.01 Å, while following simulation the sharpness of the peak was no longer there and a lower distribution was seen. During the simulation, the bond was stronger.

#### 3.8.2. RDF of PPARγ complexes

The hydrogen bonds in PPARγ complexes depicted the tight bound of active cavity residue with the ligand over the timescale of simulation. Binding site residues were mainly involved in making hydrogen bonds with the ligand within the different time scales. The residues Arg82:NE, Arg:NH2 and Ser136:N were subject to RDF as these residues were playing a critical role in the stability of Raltitrexed inside the binding cavity of PPARγ (Fig 17(C)). The Arg82:NE atom of PPARγ was simultaneously involved in the hydrogen bonding with two atoms (O6 and O7) of ligand. The RDF of Arg82:NE with O6 showed a sharp distribution peak with the g (r) value of 1.1 at 100 ns whereas, with O7, the sharp peak of the g (r) value of 1.0 was observed at 200 ns. The other atom (NH2) of Arg82 was also engaged in a strong hydrogen bond with the O7 of the ligand. Initially, it showed the g (r) value of 0.31 at 2.7 Å whereas at 100 ns the peak become narrower and showed maximum distribution. The value of g (r) highest peak at 100 ns with the g(r) of 0.95 at the distance of 2.7 Å which again decreased at the end of simulation having the value for g(r) of 0.48 at the 2.9 Å. Residue Ser136:N interacting with O3 atom of ligand showed the g(r) value of 0.75 and 0.60 at 2.9 Å before and after simulation respectively.

In Lanifibranor-PPARγ, the RDF for Lys161:NZ was calculated as it was strongly involved in the hydrogen bond (Fig 16(D)). Between the ligand and protein, a steep peak with a g(r) value of 1.3 at the start and 1.4 at the end of simulation was seen, however at 100 ns, the g(r) value was 0.87 with no evident sharp peak identified. All peaks were observed at the same distance of 2.7 Å (Fig 17(D)). The value of gyration at the end of the simulation was very close to the starting peak. This shows that the ligand was moving closer in the active cavity of the protein in the timespan of 200 ns simulation and was tightly bound at its active site till the end of the simulation.

#### 3.8.3. RDF of PPARδ complexes

Hydrogen bond analysis revealed the active residues of the PPARδ (GLN250 and THR256 of Iloprost-PPARδ, Arg248, and Tyr437 of Lifitegrast-PPARδ and ILE327 and THR253 of Lanifibranor-PPARδ) were found as key residues involved in strong intermolecular interactions. An RDF graph was generated for interacting residues with ligands for further investigation. In the Iloprost-PPARδ complex, GLN250:NE2 was making two hydrogen bonds. At the beginning of the simulation, the highest peak for GLN250:NE2 and atom O6 the ligand appeared at 2.7 Å and g(r) value was 2.59, atom O8 of the ligand was also found interacting with GLN250:NE2 value 4.7 Å having the g(r) value 0.40. At time 100 ns, the peak for GLN250:NE2 with O6 and O8 of the ligand appeared at 4.3 Å and 2.8 Å with the value of g (r) 0.30 and 2.0 respectively whereas finally at the end of simulation GLN250:NE2 interacting with ligand, distributed to a maximum value of g(r) value 1.02 for O6 and 1.44 for O8 of the ligand at 2.7 Å. It was observed that the peak was high initially. The other residue THR256:OG interacting with the O4 of the ligand, at the start of the simulation showed a maximum distribution peak at 2.9 Å and the g(r) value was 1.21. At 100 ns the distribution peak was observed for THR256:OG1 and atom O4 of the ligand at 3.6 Å with the g(r) of 0.55. Finally, at the 200 ns time scale, the distribution peak for the same interaction was observed at 3.5 Å having the g (r) value of 0.46. The distribution peak in the graph shows that the peak was narrow in width initially than at the end of the simulation.

In the Lifitegrast-PPARδ complex, hydrogen bond analysis showed that ARG248 was forming two hydrogen bonds (Fig 17(E)). ARG248:NE interaction with the O27 of the ligand showed the same distribution i-e: 2.8 Å showing the g(r) value 1.01 at the start and the end of simulations while at 100 ns this interaction showed the same distribution of 2.8 Å but with the g (r) value 0.75. The highest peak was observed for ARG248:NH2 and O27 of ligand at 2.8 Å with a g (r) value of 0.42. No specific change in this interaction was observed during the whole course of the simulation, the value of g (r) was observed 0.45 and 0.59 at 100 ns and 200 ns respectively. The interaction of TYR437: OH with O3 of the ligand showed the maximum peak at 3.3 Å revealing g(r) of 0.20 initially at the simulation, at 100 ns the peak increased and the distance decreased to 2.7 Å with g(r) 1.13 then a minor change of g(r) 1.15 at 2.6 Å was observed at the end of the simulation. The decrease in the distance shows that the ligand tries to move closer to the active cavity. In Lanifibranor-PPARδ THR253:OG1 formed the strongest bond with O5 of the ligand (Fig 17(F)). At different timescales during the simulation highest peak was observed at the same distribution i-e: 2.6 Å with the g (r) of 2.66, 2.28, and 2.63 at 0 ns, 100 ns, and 200 ns respectively showing almost little or no change. The continuous high peak shows that the ligand is highly stable in the active cavity.

### 3.9. Binding free energy calculation

The binding free energy calculations are used to determine the strength of ligand-protein binding affinities [82]. It is a powerful tool in rational drug design. In drug designing, the most common and reliable end-point techniques for estimating binding free energy are MM/GBSA and MM/PBSA. All PPAR complexes were elucidated for binding free energies using the MM/GBSA and MM/PBSA approach of AMBER.

#### 3.9.1. BFEC of PPARα complexes

Table 5 summarizes the binding energies of APHM13, Forasartan, and Lanifibranor to PPARα from MM/GBSA and MM/PBSA. The Van der Waals energy was the favorable energy calculated for all the PPARα complexes, ranging from (-33.5590 to -58.9482 kcalmol^-1^) same for both Poisson-Boltzmann (PB) and Generalized Born (GB). The difference of binding energies between GB and PB is dependent on the polar solvation energy (EPB/GB) which ranges between (0.2471 to 50.2175 kcalmol^-1^) from PB and between (0.9022 to 53.3592 kcalmol^-1^) from GB. The total binding energies for APHM13-PPARα, Forasartan-PPARα and Lanifibranor-PPARα from MM/PBSA were -36.1840 kcalmol-1, -37.5329 kcalmol-1 and -23.0744 kcalmol-1 respectively and from MM/GBSA, it was -45.0078 kcalmol-1, -36.5399 kcalmol-1 and -22.5616 kcalmol-1. All complexes were stable whereas the APHM13 and Forasartan showed higher binding affinity with PPARα.

**Table 5:** The binding energies of MMPBSA and MMGBSA for the PPARα complexes.

Energy Component	Energy Values (kcalmol^-1^)					
	APHM13-PPARα		Forasartan-PPARα		Lanifibranor-PPARα	
	GB	PB	GB	PB	GB	PB
**VDWAALS**	-58.9482	-58.9482	-56.2629	-56.2629	-33.5590	-33.5590
**EEL**	-4.2216	-4.2216	-26.0858	-26.0858	13.2301	13.2301
**EGB/PB**	25.6807	32.9698	53.3592	50.2175	0.9022	0.2471
**ESURF**	-7.5187	N/A	-7.5503	N/A	-3.1349	N/A
**ENPOLAR**	N/A	-5.9840	N/A	-5.4017	N/A	-2.9926
**EDISPER**	N/A	0.0000	N/A	0.0000	N/A	0.0000
**DELTA G gas**	-63.1698	-63.1698	-82.3487	-82.3487	-20.3289	-20.3289
**DELTA G solv**	18.1620	26.9858	45.8089	44.8158	-2.2327	-2.7455
**DELTA TOTAL**	-45.0078	-36.1840	-36.5399	-37.5329	-22.5616	-23.0744

#### 3.9.2. BFEC of PPARγ complexes

The total binding energy for PPARγ in complex with Raltitrexed and Lanifibranor were -26.4731 kcalmol^-1^ and -39.8324 kcalmol^-1^ respectively, from PB calculations whereas from GB it was -26.8237 kcalmol^-1^ for with Raltitrexed and -43.0828 kcalmol^-1^ for Lanifibranor. The favorable Van der Waals energy for PPARγ-Raltitrexed and PPARγ-Lanifibranor from both PB and GB were-43.4097 kcalmol^-1^ and -47.1082 kcalmol^-1^. The binding energy calculations of Raltitrexed in complex with PPARγ showed higher binding energy from both methods which depict the stability and strong binding potency between receptor and ligand molecules. The binding energies of all PPARγ complexes are shown in Table 6.

**Table 6:** The binding energies of MMPBSA and MMGBSA for the PPARγ complexes.

Energy Component	Energy Values (kcalmol^-1^)			
	Lanifibranor-PPARγ		Raltitrexed-PPARγ	
	GB	PB	GB	PB
**VDWAALS**	-43.409	-43.409	-47.108	-47.108
**EEL**	-34.413	-34.413	-81.989	-81.989
**EGB/PB**	56.889	56.007	92.839	94.611
**ESURF**	-5.889	N/A	-6.825	N/A
**ENPOLAR**	N/A	-4.657	N/A	-5.346
**EDISPER**	N/A	0.000	N/A	0.000
**DELTA G gas**	-77.823	-77.823	-129.097	-129.097
**DELTA G solv**	50.999	51.350	86.014	89.264
**DELTA TOTAL**	-26.823	-26.473	-43.082	-39.832

#### 3.9.3. BFEC of PPARδ complexes

In MM/GBSA and MM/PBSA, the PPARδ complexes have binding energy in the range between (-36.7277 to -45.4016 kcalmol^-1^) for PB where the GB range is between (-27.3368 to -45.0213 kcalmol^-1^). The formation of PPARδ complexes results were highly favorable for coulombic interactions during calculations. The electrostatic contributions were in the range between (-102.5498 to -166.2902 kcalmol^-1^) for PB and GB both. The PB and GB values for Iloprost-PPARδ, Lifitegrast-PPARδ, and Lanifibranor-PPARδ are listed in Table 7.

**Table 7:** The binding energies of MMPBSA and MMGBSA for the PPARδ complexes.

Energy Component	Energy Values (kcalmol^-1^)					
	Iloprost-PPARδ		Lifitegrast-PPARδ		Lanifibranor-PPARδ	
	GB	PB	GB	PB	GB	PB
**VDWAALS**	-50.738	-50.738	-69.476	-69.476	-45.416	-45.416
**EEL**	-102.549	-102.549	-166.290	-166.290	-164.892	-164.892
**EGB/PB**	115.656	121.569	200.031	196.637	189.237	173.941
**ESURF**	-7.389	N/A	-8.670	N/A	-6.265	N/A
**ENPOLAR**	N/A	-5.009	N/A	-6.273	N/A	-4.126
**EDISPER**	N/A	0.000	N/A	0.000	N/A	0.000
**DELTA G gas**	-153.287	-153.287	-235.766	-235.766	-210.308	-210.308
**DELTA G solv**	108.266	116.560	191.361	190.364	182.972	169.815
**DELTA TOTAL**	-45.021	-36.727	-44.405	-45.401	-27.336	-40.493

### 3.10. Principal component and free energy landscape analysis

**Fig 18:**
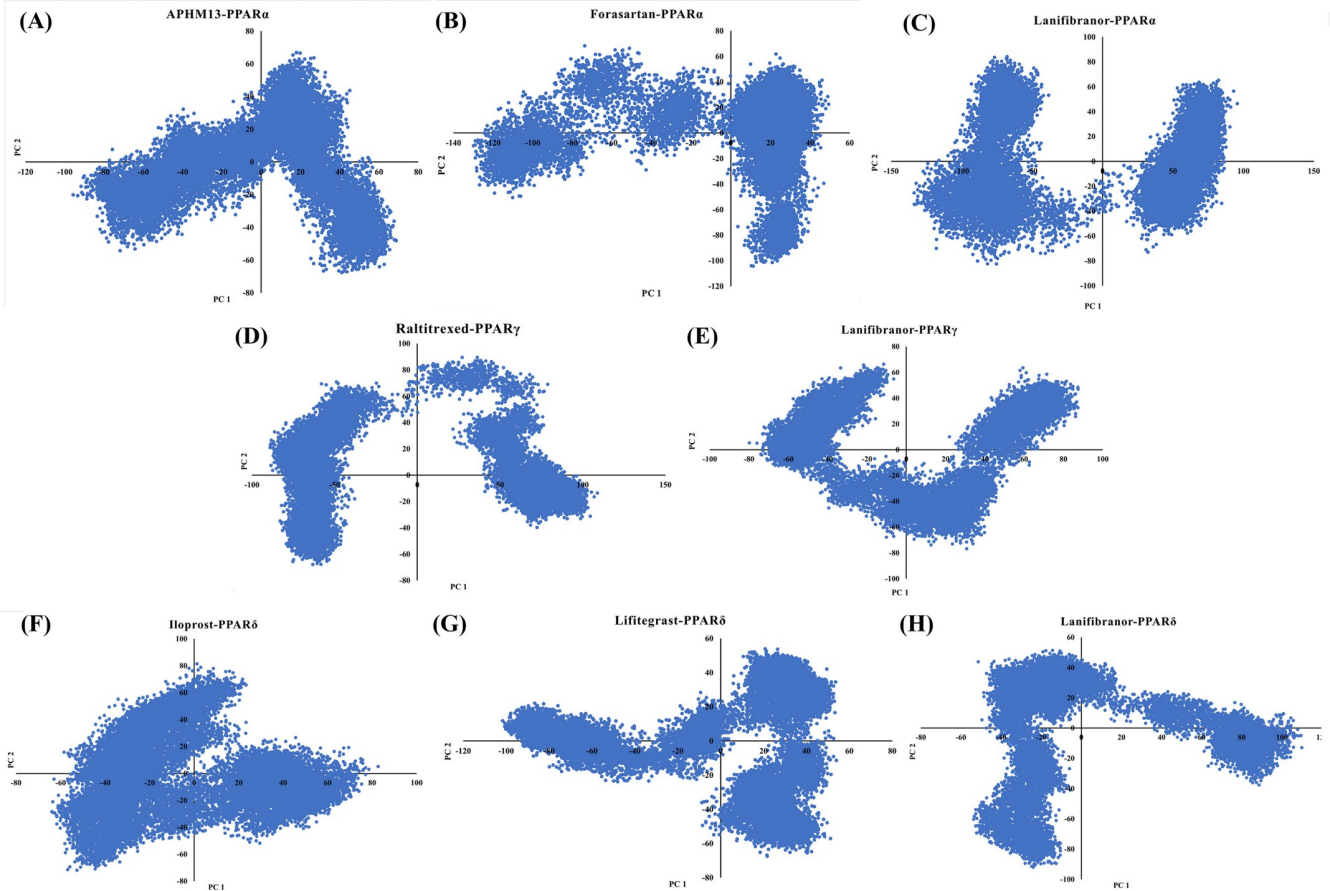
Constructed Principal component analysis (PCA) for (A) PPARα-APHM13, (B) PPARα-Forasartan, (C) PPARα-Lanifibranor, (D) PPARγ-Raltitrexed, (E) PPARγ-Lanifibranor, (F) PPARδ-Iloprost, (G) PPARδ-Lifitegrast, (H) PPARδ-Lanifibranor.

Numerous internal movements of the protein molecules are hard to comprehend. Principal component analysis (PCA) is a method for reducing the massive dimensions of a data set to the primary principal components, displaying the key variations that would represent the protein’s global motion with the crucial information. The PCs obtained during MD simulation are derived from the eigenvector values of the covariance matrix, each of which correlates to a variation in protein trajectories. PCA was employed to analyze the dynamics of protein-ligand complexes in order to better understand the influence of ligands binding on protein dynamics. It can be seen from Fig 18, that the clusters of APHM13-PPARα were more compact and the movements were confined to smaller space whereas the projection of the Forasartan-PPARα and Lanifibranor-PPARα captured more essential subspace. The distortion in the projection of Lanifibranor-PPARα system showed the high flexibility of the Ω-loop during the simulation which aided in protein ligand interaction, compliance with RMSD and RMSF results (Fig 10). This depicts how the Lanifibranor increased the internal movements due to the interaction between Lanifibranor and PPARα, prompting PPARα to adopt a new configuration with a smaller subspace. The U-shaped projection of Lanifibranor-PPARγ demonstrates the rise and decrease in mobility around the protein backbone whereas the compactness of the clusters indicates the stability of the system. The PCA plot for Raltitrexed-PPARγ depicted a minor distortion which was due to a structural change in the Ω-loop (Fig 13). The motions of the Iloprost-PPARδ were confined to a lesser space and the clusters showed compactness which indicated the system was highly stable. The compactness in the cluster projections was also observed in Lifitegrast-PPARδ and Lanifibranor-PPARδ systems but takes wider space. It implies that the binding of Lifitegrast and Lanifibranor to PPARδ promotes internal motion due to their strong interaction.

**Fig 19:**
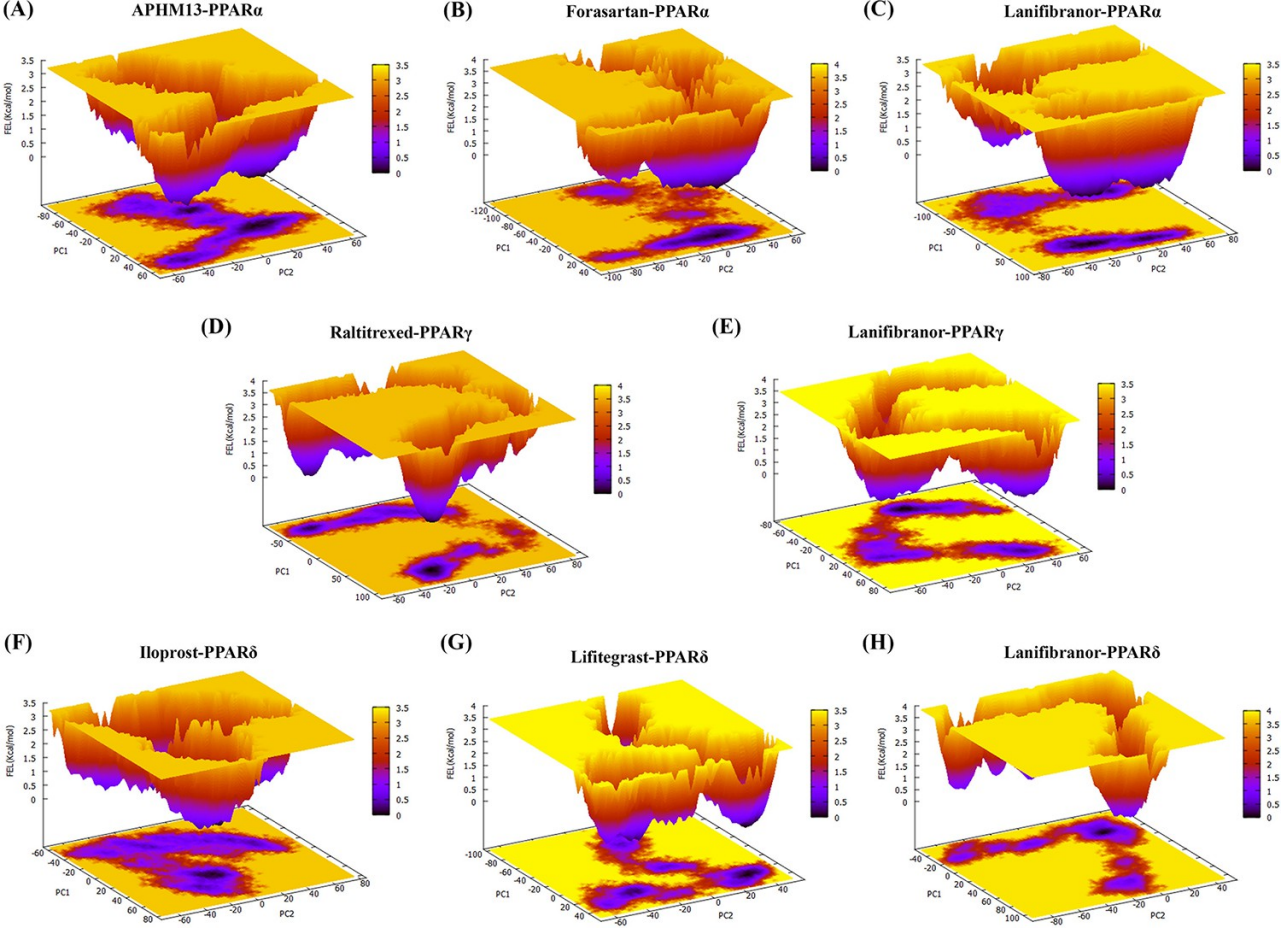
FEL is calculated as a function of MD trajectory projections onto the first (PC1) and second (PC2) eigenvectors, respectively. (A) PPARα-APHM13, (B) PPARα-Forasartan, (C) PPARα-Lanifibranor, (D) PPARγ-Raltitrexed, (E) PPARγ-Lanifibranor, (F) PPARδ-Iloprost, (G) PPARδ-Lifitegrast, (H) PPARδ-Lanifibranor.

The free energy landscape (FEL) approach, which is based on PCA, provides a more accurate depiction of the protein conformational space in terms of energy and time. Fig 19 illustrates the free energy landscapes projected onto the first two principal components of all complexes for the backbone atoms of the proteins. The Lanifibranor and the FDA-approved drugs have far more stability, as evidenced by the size and form of the minimal energy area (black) in the free energy contour map. Orange regions that are smaller and more concentrated imply that the respective complex is more stable. All the ligands binding to their respective receptors tend to reach thermodynamically stable conformation. The results show that these agonists offer favorable conformational change towards the PPARs.

### 3.11. Dynamic cross correlation matrix

**Fig 20:**
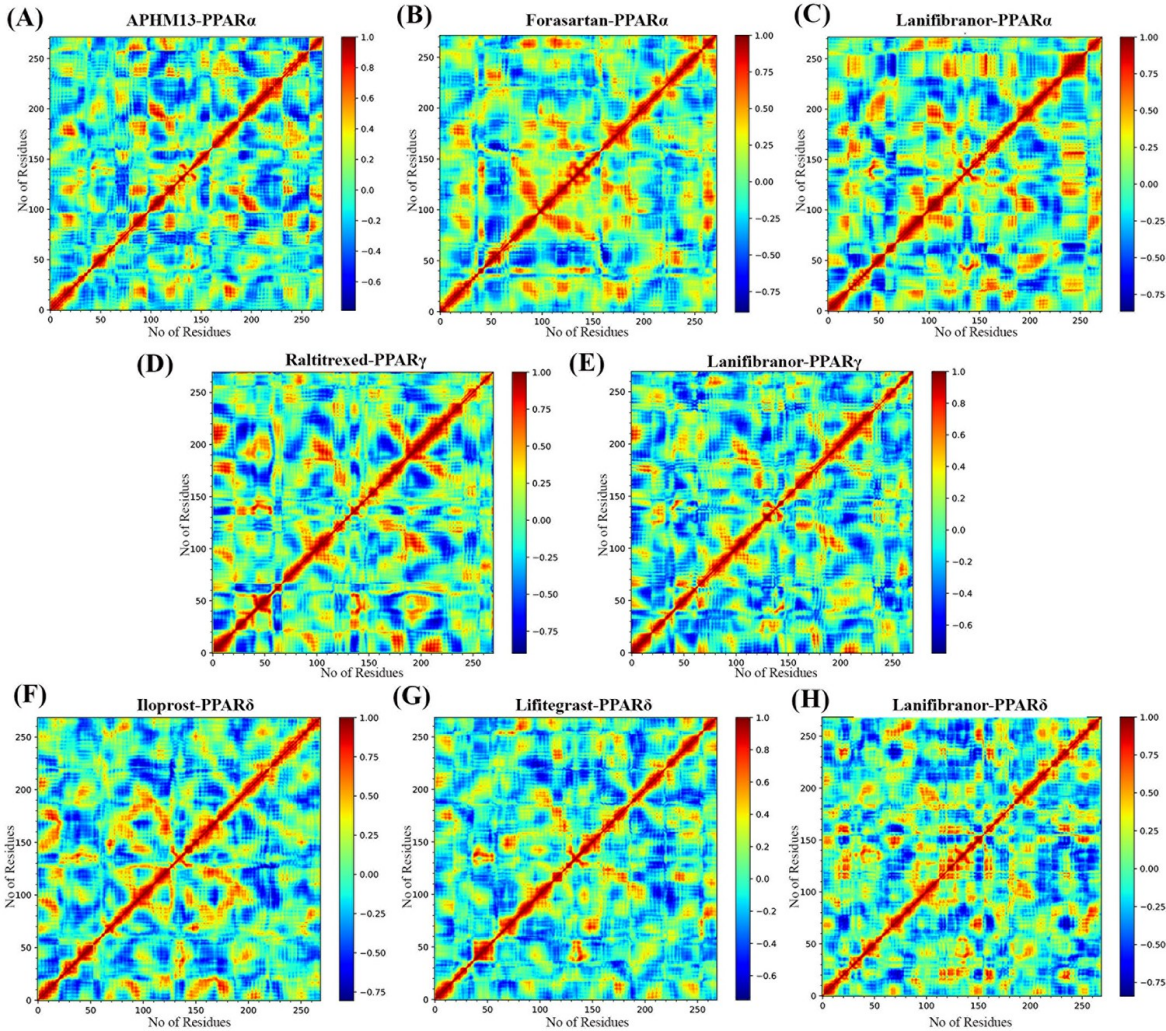
The dynamic cross-correlation matrix of backbone atoms throughout the simulation duration of 200-ns. (A) PPARα-APHM13, (B) PPARα-Forasartan, (C) PPARα-Lanifibranor, (D) PPARγ-Raltitrexed, (E) PPARγ-Lanifibranor, (F) PPARδ-Iloprost, (G) PPARδ-Lifitegrast, (H) PPARδ-Lanifibranor.

Dynamic Cross-correlation matrix was constructed using the coordinates of Cα atoms from MD trajectories to illustrate the impact of agonists binding on the internal dynamics of PPARs, and the dynamic cross-correlation map (DCCM) is shown in Fig 20. In the case of PPARα complexes, the increase in correlation motion was observed in Forasartan-PPARα and Lanifibranor-PPARα especially in the Ω-loop region and in the region around it. The maps also revealed the diagonals to be highly correlated with a particular probability of similar conformational changes. The Raltitrexed-PPARγ map represents the rise in both correlated and anti-correlated motion while the anti-correlation motion on the map for Lanifibranor-PPARγ was minimized. This revealed the major differences occurred in specific regions of PPARγ in complex with Raltitrexed. Compared to the Iloprost- PPARδ system, the correlation map for Lifitegrast- PPARδ showed a little decrease in correlation motion. The Lanifibranor-PPARδ exhibited a similar correlation to the Iloprost-PPARδ, confirming that the positive correlation may be attributable to the adopted confirmation of the PPARδ. These observations of ligand-induced conformational changes reveal the significance of internal dynamics in activation of PPARs.

### 3.12. Role of AF-2 domain

The H-12 forms a large portion of the AF-2 surface since stabilization of H-12 allows the receptor to heterodimerize with the retinoid X receptor (RXR) and allows the recruitment of coactivators for PPAR regulated target gene transcription [83]. From the literature, it was determined that Lanifibranor is a Pan PPAR agonist that fully activates PPARα and PPARδ while partially activating PPARγ [43]. The computational analysis agrees with the outcome obtained from clinical trials since MD simulations demonstrated that Lanifibranor showed interaction with H-12 in PPARα and PPARδ whereas it lacked interaction with H-12 in PPARγ, resulting in the partial activation of PPARγ. During simulation, inward movement of Ω-loop brought Lanifibranor closer to the H-12 of PPARα (interacting with Pro262 and Gln265 residues) and provided proximity to bound ligands, which might play a crucial role in ligand-receptor interactions [76–78]. Whereas Lanifibranor formed bonds (residues Thr253, His287, His413, and Tyr437) with H-12 of PPARδ, which are crucial for stabilizing the AF-2 domain [79]. The structural assistance for co-activator recruitment is provided by the stability of the AF-2 domain in the active helical state.

Forasartan showed interactions with two critical residues: Ser84 of H-3 and His244 of H-10, both of which play essential roles in the agonist-induced activation of PPARα [79]. Indirectly, this interaction between H-3 and loop 11–12 contributes to the further stabilization of H-12’s active conformation. Raltitrexed and Lifitegrast followed the same binding pattern as Lanifibranor in PPARγ and PPARδ respectively. Lifitegrast was stabilizing the AF-2 domain by interacting with the crucial residues of H-12 in PPARδ whereas Ralititrexed devoid interaction with H-12 in PPARγ. In the case of the PPARγ-Raltitrexed complex, the extension and contraction of H-12 were also detected. This might be due to the ligand’s lack of direct interaction with H-12. When H-12 of the complex was not stabilized by the ligand, the overall fluctuations in the complex rise [83]. Upon mapping top-scored FDA-approved drugs on their respective pharmacophore, it was revealed that Forasartan and Lifitegrast mapped nicely with all essential features of pharmacophore, on the other hand, one HBA failed to map on Raltitrexed same as Lanifibranor. Since no PPARγ’s ligands were found to be interacting with the H-12 and both missed one pharmacophoric feature (HBA), assuming in this way a character of partial agonist. When compared to full agonists, partial agonists are weak activators of PPAR that evoke the same activation pattern and have connected dose-response curves with reduced transactivation potential [84]. Considering over-activation of PPARγ might result in significant adverse effects such as weight gain and steatosis, PPARγ partial agonists are preferable [84]. This study might facilitate in designing of balanced drugs for PPARs in the future, eliminating the side effects seen with PPARγ full agonist currently available in markets which is a major challenge for pharmaceutical firms.

## 4. Conclusion

This study performed a series of computer-aided structural techniques to explore therapeutic potential of the third phase clinical trial PPAR pan-agonist; Lanifibranor. Molecular dynamic studies of Lanifibranor showed that it can be a promising drug candidate in treating NASH, that fully activates PPARα, and PPARδ whereas partially activate PPARγ. Moreover, FDA-approved drugs: Forasartan, Raltitrexed, and Lifitegrast also stand out as potential agonists for PPARα (full agonist), PPARγ (partial agonist), and PPARδ (full agonist), respectively. Lanifibranor facilitates compact AF-2 Domain organization which in turn assists the attachment of co-activator. The PPARγ agonist Raltitrexed and Lanifibranor exhibited as partial activators due to the lack of a pharmacophore feature (HBA) leading to the loss of H-12 interactions. Furthermore, this study has also led to the identification of common chemical scaffolds (methyl sulfonyl benzene, butyric acid, and chlorobenzene) which provide a good starting point for designing new drugs against PPARs. These drugs can also be considered in combinational therapy against NASH followed by *in vitro* and *in vivo* studies to determine the practical function of combination treatment. This study will facilitate in designing of balanced drugs for PPARs in the future, eliminating the side effects seen with PPARγ full agonist currently available in markets which is a major challenge for pharmaceutical firms.

## Supporting information

S1 Table The ligands used in training set for PPARα with their structure and EC50 value. (DOCX)

S2 Table The ligands used in training set for PPARγ with their structure and EC50 value. (DOCX)

S3 Table The ligands used in training set for PPARδ with their structure and EC50 value. (DOCX)

S1 Graphical abstract (TIF)
