# Supplementary material for: Deciphering the relational dynamics of AF-2 domain of PAN PPAR through drug repurposing and comparative simulations
Source: PLoS One. 2023 Mar 31;18(3):e0283743. doi: 10.1371/journal.pone.0283743 (PMC10065303; doi:10.1371/journal.pone.0283743)
Supplement: S1 Table — (DOCX) [file pone.0283743.s001.docx]

**Supporting Information**

**S1 Table.** The ligands used in training set for PPARα with their structure and EC50 value.

| **S.no** | **Ligands** | **EC50 (µM)** | **Structure** |
| --- | --- | --- | --- |
| 1 | 1 | 0.001 |  |
| 2 | 2 | 0.0013 |  |
| 3 | GW409544 | 0.002 |  |
| 4 | Aliglitazar | 0.005 |  |
| 5 | TIPP-401 | 0.01 |  |
| 6 | 3 | 0.01 |  |
| 7 | 4 | 0.0113 |  |
| 8 | 5 | 0.012 |  |
| 9 | 6 | 0.03 |  |
| 10 | LY518674 | 0.042 |  |
| 11 | NS-220 | 0.042 |  |
| 12 | Elafibranor | 0.045 |  |
| 13 | GW9578 | 0.05 |  |
| 14 | TIPP-703 | 0.061 |  |
| 15 | Imiglitazar | 0.067 |  |
