# Supplementary material for: Deciphering the relational dynamics of AF-2 domain of PAN PPAR through drug repurposing and comparative simulations
Source: PLoS One. 2023 Mar 31;18(3):e0283743. doi: 10.1371/journal.pone.0283743 (PMC10065303; doi:10.1371/journal.pone.0283743)
Supplement: S2 Table — (DOCX) [file pone.0283743.s002.docx]

**Supporting Information**

**S2 Table**. The ligands used in training set for PPARγ with their structure and EC50 value.

| S.No | Ligands | Ec50(µM) | Structure |
| --- | --- | --- | --- |
| 1 | GW409544 | 0.00028 |  |
| 2 | Saroglitazar | 0.003 |  |
| 3 | Calbiochem (Gw1929) | 0.0062 |  |
| 4 | S26948 | 0.008 |  |
| 5 | Aleglitazar | 0.009 |  |
| 6 | Imilitazar | 0.031 |  |
| 7 | Compound40 | 0.034 |  |
| 8 | Rosiglitazone | 0.034 |  |
| 9 | TIPP-703 | 0.043 |  |
| 10 | Compound35 | 0.091 |  |
