# Supplementary material for: Deciphering the relational dynamics of AF-2 domain of PAN PPAR through drug repurposing and comparative simulations
Source: PLoS One. 2023 Mar 31;18(3):e0283743. doi: 10.1371/journal.pone.0283743 (PMC10065303; doi:10.1371/journal.pone.0283743)
Supplement: S3 Table — (DOCX) [file pone.0283743.s003.docx]

**Supporting Information**

**S3 Table**. The ligands used in training set for PPARδ with their structure and EC50 value.

| **S.No** | **Ligands** | **EC50(µM)** | **Structures** |
| --- | --- | --- | --- |
| 1 | TIPP-204 | 0.0009 |  |
| 2 | Endurobol (GW501516) | 0.001 |  |
| 3 | GW0742 | 0.001 |  |
| 4 | L-1650471 | 0.002 |  |
| 5 | Seladelpar  (MBX-8025) | 0.002 |  |
| 6 | GNF-0242 (ZINC13015184) | 0.004 |  |
| 7 | GNF-8065  (ZINC58163616) | 0.006 |  |
| 8 | GNF-8501  (ZINC23642332) | 0.006 |  |
| 9 | GNF-3632  (ZINC11484008) | 0.007 |  |
| 10 | GNF-6878  (ZINC03242660) | 0.008 |  |
| 11 | GNF-0341  (ZINC12975568) | 0.011 |  |
| 12 | GNF-8560  (ZINC09587215) | 0.011 |  |
| 13 | GNF-6029  (ZINC23257925) | 0.012 |  |
| 14 | TIPP-401 | 0.012 |  |
| 15 | GNF-5891  (ZINC10292903) | 0.013 |  |
| 16 | GNF-5295  (ZINC20218763) | 0.017 |  |
| 17 | GNF-7486  (ZINC09635461) | 0.018 |  |
| 18 | DB959 | 0.019 |  |
| 19 | GNF-6952  (ZINC48278659) | 0.019 |  |
| 20 | TIPP-703 | 0.12 |  |
