# Supplementary figures and images for: Deciphering the relational dynamics of AF-2 domain of PAN PPAR through drug repurposing and comparative simulations
Source: PLoS One. 2023 Mar 31;18(3):e0283743. doi: 10.1371/journal.pone.0283743 (PMC10065303; doi:10.1371/journal.pone.0283743)

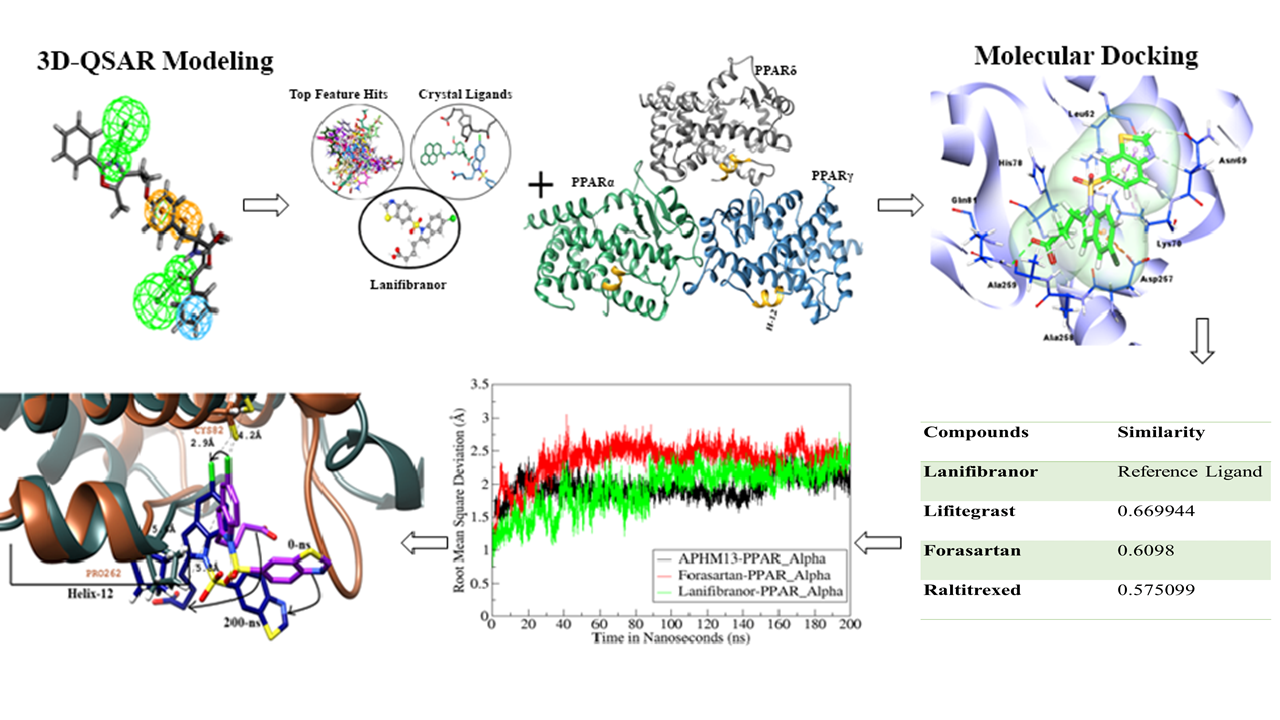

Supplement: S1 Graphical abstract — (TIF) [file pone.0283743.s004.tif]
